# Figure-of-eights vs running suture for fascial closure of large ventral hernias: do figure-of-eights induce ischemia?

**DOI:** 10.1007/s00464-026-13036-5

**Published:** 2026-06-30

**Authors:** William C. Bennett, Alvaro C. Carvalho, Emerson Lora, Erika M. Schmidt, Noah X. Tocci, Ashley M. Mila-Hoff, Joseph Edwards, Cassandra Hennessy, Luciano G. Tastaldi, Lucas R. Beffa, David M. Krpata, Ajita S. Prabhu, Clayton C. Petro, Benjamin T. Miller

**Affiliations:** 1 Center for Abdominal Core Health, Cleveland Clinic Foundation, 2049 E 100th St A Bldg, A10-133, Cleveland, OH 44195 USA; 2 Department of Biostatistics, Vanderbilt University, Nashville, TN USA; 3 Cleveland Clinic Lerner College of Medicine at the Case Western Reserve University School of Medicine, Cleveland, OH USA

**Keywords:** Ventral hernia repair, Running suture, Figure-of-eight, Fascial closure, Herniorrhaphy

## Abstract

**Introduction:**

Many techniques can reapproximate fascia during herniorrhaphy. Reports suggest utility of interrupted figure-of-eights for large defects less amenable to running suture closures. However, anecdotal concerns regarding ‘ischemic’ qualities of figure-of-eights may dissuade utilization, despite no substantiating evidence. This study compares wound morbidity for figure-of-eights vs running sutures for fascial closure during open ventral hernia repair (VHR).

**Methods:**

A prospectively maintained hernia-specific registry was queried for open VHRs from 1/1/2014 to 12/31/2023 with fascial closure, ≥ 10 cm defect width, CDC Class 1 wound, fascial closure via figure-of-eights or running suture, and clinical follow-up at 30 days. Cases featuring anterior myofascial or cutaneous flaps were excluded. Surgical site occurrence (SSO), surgical site infection (SSI), SSO/SSI requiring procedural intervention (SSO/I-PI), recurrence, and patient-reported quality-of-life (QoL) at 30-day and 1-year intervals were considered. SSO at 30 days was selected as the primary outcome to serve as a surrogate for suture line ischemia, and 1-year recurrence was a key secondary outcome of interest. Baseline factors including age, BMI, gender, race, ASA class, immunosuppressant use, smoking, diabetes, hernia width, and hernia length were controlled via multivariable regression.

**Results:**

A total 4195 cases met criteria and 31% utilized figure-of-eights (*n* = 1317). Multivariable logistic regression found no increased risk of 30-day SSO or 1-year recurrence for figure-of-eight vs running suture closure. Adjusted odds of SSI (OR 1.47; 95% CI 1.11, 1.95) and SSO/I-PI (OR 1.31; 1.02, 1.70) were greater for figure-of-eight closures. No associations were identified regarding patient-reported QoL or 1-year outcomes.

**Conclusions:**

This retrospective analysis of wound morbidity in clean cases identified an increased risk of SSI and SSO/I-PI for figure-of-eight closures at 30 days vs running closures. Though the association may relate to residual confounding from selective utilization in high complexity repairs rather than an ischemic nature, the results suggest figure-of-eights should perhaps be reserved for challenging or high-tension fascial reapproximation.

**Graphical abstract:**

**Figure:**
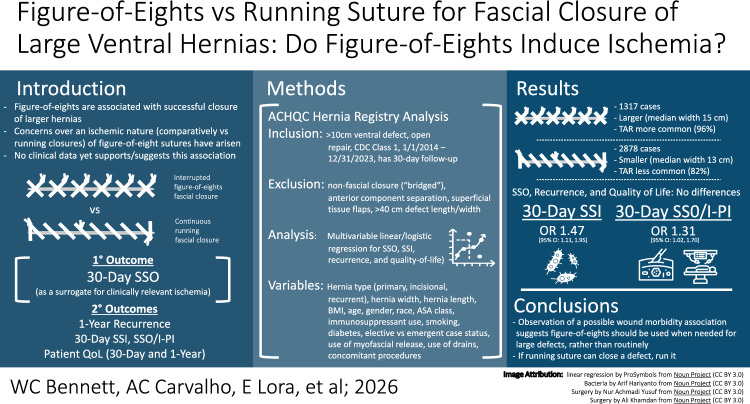

**Supplementary Information:**

The online version contains supplementary material available at 10.1007/s00464-026-13036-5.

A variety of suture techniques reapproximate fascia during incisional herniorrhaphy. Reapproximation of the linea alba via interrupted figure-of-eight sutures is often employed at our center, particularly for larger defects and during posterior component separation [1, 2]. Continuous running sutures are also frequently (and more commonly) employed [3]. Limited outcomes data substantiate superiority of one over the other, leaving the choice to surgeon preference. As limited data exist in the context of hernia repair, preferences for running suture appear to originate from extrapolations of primary laparotomy literature, including theoretically improved distribution of tension along the suture line and data demonstrating decreased operative time for standard midline laparotomy closure [4]. Notably, however, widely held perceptions of running suture superiority for primary laparotomy closure are less supported than realized. In a reversal of the “strong” recommendation provided in 2015, the updated 2022 European/American Hernia Society guidelines for primary laparotomy closure’s recommendation for continuous suture fascial closure were designated as “weak” and based on low quality evidence [5, 6].

In the context of complex ventral herniorrhaphy, however, recent data support figure-of-eight suture for midline fascial closure, suggesting interrupted figure-of-eight utilization is associated with odds of successful primary fascial closure for defects ≥ 15 cm [7]. The finding was met cautiously due to theoretical concerns of ‘ischemic’ qualities of figure-of-eight stitches—presumed to compromise wound healing and repair integrity. These concerns are likely related to the technique’s historical utilization for hemostasis, vessel ligation, and capillary tamponade [8–10]. Surprisingly limited literature provides insight into the veracity and clinical significance of this commonly held concern.

The present study assesses wound morbidity outcomes as surrogates for clinically relevant suture line ischemia. We hypothesize no differences will be observed in early surgical site occurrences (SSO) or 1-year recurrence events for figure-of-eight vs running closures during open ventral hernia repair (VHR).

## Materials and methods

After obtaining Institutional Review Board (IRB) approval, the Abdominal Core Health Quality Collaborative (ACHQC) registry was queried for cases from January 1, 2014 through December 31, 2023 featuring adult patients with clinical 30-day postoperative follow-up after open ventral hernia repair (VHR) and met the following inclusion criteria: ≥ 10 cm defect width, complete midline fascial closure, Center for Disease Control (CDC) Class 1 wound, only one fascial closure method documented, and fascial closure done via figure-of-eight or running suture. Cases featuring anterior component separation (traditional or endoscopic), free flaps, rotational flaps, and other skin/subcutaneous flaps were excluded to control for wound morbidity associated with undermining flaps [11, 12]. Hernias with recorded widths or lengths > 40 cm after application of eligibility criteria were censored as likely data entry errors (i.e., 45 cm rather than 4.5 cm). While hernias of this size are possible, such defects are unlikely to have accomplished complete midline fascial closure to meet inclusion criteria [13]. The ACHQC is an international hernia-specific registry of prospectively entered clinical data by surgeons. Details of the database have been reported elsewhere [14]. Within the specified study period, 142,289 cases from 515 surgeons were recorded in the registry.

Demographic, medical, and operative factors include age, body mass index (BMI, mg/kg^2^), patient-reported gender, race, American Society of Anesthesiologist (ASA) class, present use of immunosuppressant medication, diabetes mellitus (DM), smoking within one month of operation, hernia etiologic factors (i.e., primary, incisional, and/or recurrent), hernia width, hernia length, elective case status, use of myofascial release, use of drains, and concomitant procedure(s). Age, BMI, hernia width, and hernia length were treated as continuous variables in regression modeling; other variables were recorded in a binary or categorical fashion, as appropriate. Postoperative outcomes were considered at 30-day (14–45 days) and 1-year (8–18 months) follow-up intervals. Clinical outcomes included SSO, surgical site infection (SSI), SSO/SSIs requiring procedural intervention (SSO/I-PI), recurrence, and reoperations related to the prior hernia repair (“reoperations” unless otherwise specified).

Wound morbidity via SSO at 30 days—defined as any clinician-reported instance of twenty-two prespecified surgical site complications including fascial disruption, skin or soft tissue ischemia, skin or soft tissue necrosis, and exposed mesh—was selected as the principal outcome of interest (full list in Supplemental Table 1). SSO was selected by the research group as the best available wound morbidity outcome to act as a surrogate for suture line perfusion inadequacy, though this association is theoretical, indirect, and not validated. Recurrence at 1-year was prespecified as a key secondary outcome of interest to detect signals relating to differential recurrence patterns between figure-of-eight and running suture fascial closures.

SSO, recurrence, SSI, SSO/I-PI, and reoperation were all considered at 30-day and 1-year intervals. The hernia-specific quality-of-life survey (HerQLes), Patient-Reported Outcomes Measurement Information System Scale v1.0 Pain Intensity Short-Form 3a (PROMIS 3a), and ventral hernia recurrence inventory (VHRI) bulge question were utilized to detect differential signals in abdominal wall-specific QoL, pain, or patient-experienced bulge between the two techniques at the same intervals. HerQLes is a twelve-item questionnaire validated in patients undergoing open ventral hernia repair; the scaled score ranges from 0 to 100 with a higher score indicating a higher quality of life [15, 16]. PROMIS 3a is a validated survey to capture all-cause patient-experienced pain; responses are converted to t-scores ranging from 30.7 to 71.8 where higher scores indicate more pain [17, 18].

### Statistical analysis

Baseline patient characteristics were analyzed via simple descriptive and univariate statistics. Categorical variables were summarized by counts and percentages (%) and analyzed using Chi-squared tests (or Fisher’s exact test when appropriate). Continuous variables were summarized by median and interquartile ranges (IQR1, IQR3) and analyzed using the Wilcoxon test. A multivariable logistic regression model was used to compare 30-day SSO rates between figure-of-eight and running suture groups. The model was adjusted for patient factors including hernia type (primary, incisional, or recurrent), hernia width, hernia length, BMI, age, gender, race, ASA class, immunosuppressant use, smoking, and diabetes, and surgical factors, including elective vs emergent case status, utilization of myofascial release, use of surgical drains, and the presence of concomitant procedure. There were no missing data in the variables used in the model. Secondary outcomes were adjusted for the same covariates as the primary model. Binary outcomes were modeled using logistic regression, and continuous outcomes were modeled using linear regression. All statistical analyses were completed using R software (The R Foundation, Vienna, Austria), including Hmisc and rms packages, and were considered significant at a 5% level.

## Results

Of 140,226 cases identified within the study period, 4195 met study criteria (Supplemental Fig. 1). Figure-of-eight closure accounted for 31.4% of cases (*n* = 1317). Median age was 60 years (IQR 51–67), median BMI was 33 (IQR 29–37), most patients were female (52%), ASA Class 3 was most common (72%), and most patients were white (89%) (Table 1). Median hernia width was larger for figure-of-eight vs running suture repairs (15 cm vs 13 cm, respectively), as was hernia length (23 cm vs 20 cm). There was notable attrition for the subgroup analysis of 1-year outcomes, wherein follow-up was available for ~ 38% (*n* = 1169 with complete 1-year clinical follow-up) of patients.

**Table 1:** Patient, medical, and surgical characteristics, stratified by fascial closure method

Item	Fo8 *N* = 1317	Running * N* = 2878	Combined * N* = 4195	*P*
Age, years	60 (52, 68)	60 (51, 68)	60 (51, 68)	0.3
BMI, kg/m^2^	34 (29, 37)	33 (29, 37)	33 (29, 37)	0.008
Gender, female	50% (664)	52% (1500)	52% (2164)	0.3
Race				0.001
American Indian or Alaskan Native	0% (4)	0% (8)	0% (12)	
Asian, Native Hawaiian or other Pacific Islander	0% (2)	0% (9)	0% (11)	
Black or African American	3% (46)	7% (194)	6% (240)	
Hispanic	3% (34)	2% (71)	3% (105)	
White	92% (1206)	88% (2530)	89% (3736)	
Middle Eastern	1% (10)	1% (22)	1% (32)	
Other or unknown	1% (13)	1% (24)	1% (37)	
Not indicated	0% (2)	1% (20)	1% (22)	
ASA class				< 0.001
1	0% (6)	1% (36)	1% (42)	
2	16% (206)	28% (816)	24% (1022)	
3	80% (1052)	68% (1960)	72% (3012)	
4	4% (52)	2% (60)	3% (112)	
5	0% (0)	0% (0)	0% (0)	
None assigned	0% (1)	0% (5)	0% (6)	
Immunosuppressants	8% (106)	9% (254)	9% (360)	0.4
Smoking (current within one month)	6% (77)	8% (228)	7% (305)	0.02
Diabetes mellitus	26% (345)	24% (677)	24% (1022)	0.06
Procedure category—primary ventral	0% (2)	1% (26)	1% (28)	0.006
Procedure category—incisional	99% (1305)	97% (2802)	98% (4107)	< 0.001
Recurrent	61% (797)	48% (1373)	52% (2170)	< 0.001
Elective case	98% (1296)	98% (2833)	98% (4129)	0.9
Myofascial release(s)	96% (1263)	87% (2517)	90% (3780)	< 0.001
Rives-Stoppa	4% (47)	16% (447)	12% (494)	< 0.001
TAR	92% (1215)	72% (2067)	78% (3282)	< 0.001
Bilateral^a^	96% (1163)	87% (1799)	90% (2962)	< 0.001
Unilateral^a^	4% (52)	13% (268)	10% (320)	< 0.001
Drains used	98% (1289)	95% (2728)	96% (4017)	< 0.001
Mesh position^b^				
Retrorectus or retromuscular	97% (1238)	90% (2452)	92% (3690)	< 0.001
Preperitoneal	54% (697)	38% (1023)	43% (1720)	< 0.001
Intraperitoneal	2% (30)	4% (110)	3% (140)	0.006
Concomitant procedure performed	10% (127)	18% (529)	16% (656)	< 0.001
Hernia width (cm)	15 (13, 20)	13 (11, 15)	14 (12, 17)	< 0.001
Hernia length (cm)	23 (19, 27)	20 (15, 23)	20 (15, 25)	< 0.001

Categorical variables presented as % (*n*); continuous variables presented as median (IQR1, IQR3). Categorical tests via Chi-square or Fisher’s exact test whenever counts ≤ 5. Continuous variables comparisons via Wilcoxon ranked sum test. Rives-Stoppa defined as cases featuring posterior rectus sheath releases without transversus abdominals release

*Fo8* Figure-of-eight, *BMI* body mass index, *ASA* American Society of Anesthesiologists, *TAR* transversus abdominis release

^a^Reported percentages refer to percentage of cases featuring TAR

^b^Mesh position in the ACHQC allows the selection of multiple planes; therefore, cases may be represented in multiple mesh position categories; mesh position data were only available for 1281 cases in the figure-of-eight arm and 2717 cases in the running suture arm. Percentages are based on cases with mesh data available rather than the entire cohort

### Unadjusted outcomes

A total of 574 30-day SSOs occurred and incidence were not different between figure-of-eight and running suture cohorts (15% vs 13%; *P* = 0.1). Forty-five recurrences were identified at 1-year and incidence was similar between groups (4% vs 5%; *P* = 0.3). The figure-of-eight cohort demonstrated a higher rate of SSI (9% vs 5%; *P* < 0.001) and SSO/I-PI (10% vs 7%; *P* < 0.001) vs the running suture cohort. No further differences were identified between the cohorts at 30 days, and no differences were identified for any measure at 1-year in unadjusted analyses (Table 2).

**Table 2:** Unadjusted postoperative outcomes at (A) 30-Day and (B) 1-year follow-up windows, stratified by fascial closure method

Item	N	Fo8 (*N* = 1317)	Running (*N* = 2878)	Combined * N* = 4195	*P*
(A) 30-day follow-up outcomes					
SSO	4195	15% (197)	13% (377)	14% (574)	0.1
SSI	4195	9% (112)	5% (144)	6% (256)	< 0.001
SSO/I -PI	4195	10% (132)	7% (188)	8% (320)	< 0.001
Hernia recurrence	4195	0% (0)	0% (2)	0% (2)	0.3
Re-operation	4195	2% (32)	2% (69)	2% (101)	0.9
HerQLes summary score	2190	45 (27, 70)	47 (27, 70)	47 (27, 70)	0.4
PROMIS pain 3a t-score	2237	46 (44, 52)	46 (40, 52)	46 (40, 52)	0.06
Patient-reported bulge	2073	19% (139)	19% (253)	19% (392)	0.9
(B) 1-Year follow-up outcomes					
SSO	1169	3% (11)	1% (11)	2% (22)	0.1
SSI	1169	1% (5)	1% (8)	1% (13)	0.8
SSO/I -PI	1169	2% (8)	2% (14)	2% (22)	0.9
Hernia recurrence	1104	4% (14)	5% (34)	4% (48)	0.3
Re-operation	1183	3% (14)	4% (31)	4% (45)	0.5
HerQLes summary score	1357	80 (58, 92)	82 (57, 93)	82 (57, 92)	0.2
PROMIS pain 3a t-score	1368	31 (31, 44)	31 (31, 44)	31 (31, 44)	0.7
Patient-reported bulge	1372	21% (102)	20% (180)	21% (282)	0.8

Categorical variables presented as % (*n*); continuous variables presented as median (IQR1, IQR3). Categorical tests via Chi-square or Fisher’s exact test whenever counts ≤ 5. Continuous variables comparisons via Wilcoxon ranked sum test

*Fo8* Figure-of-eight, *BMI* body mass index, *ASA* American Society of Anesthesiologists

Patient-reported hernia-specific quality of life, patient-reported pain, and patient-reported bulge sensation were not different between groups at either timepoint (Table 2).

The most common 30-day SSO was seroma for both cohorts (figure-of-eights: *n* = 69 (5.2%); running suture: *n* = 153 (5.3%)). Fewer fascial disruptions were identified in the figure-of-eight cohort (*n* = 1) than the running suture cohort (*n* = 8), but incidence was statistically non-different (0.08% vs 0.28%, respectively; *P* = 0.18). Further specifications of 30-day SSO and SSI events are provided in Table 3.

**Table 3:** Unadjusted surgical site occurrence and surgical site infection events at 30 days, stratified by fascial closure method

Outcome	Fo8 (*N* = 1317)	Running (*N* = 2878)	*P*
Wound cellulitis	51 (3.87%)	58 (2.02%)	< 0.001
Non-healing incisional wound	13 (0.99%)	34 (1.18%)	0.579
Fascial disruption	1 (0.08%)	8 (0.28%)	0.2886
Skin or soft tissue ischemia	3 (0.23%)	23 (0.80%)	0.0286
Skin or soft tissue necrosis	6 (0.46%)	30 (1.04%)	0.0558
Wound serous drainage	32 (2.43%)	55 (1.91%)	0.2739
Wound purulent drainage	5 (0.38%)	17 (0.59%)	0.3798
Chronic sinus drainage	0 (0.00%)	1 (0.03%)	1
Localized stab wound infection	0 (0.00%)	2 (0.07%)	1
Stitch abscess	1 (0.08%)	7 (0.24%)	0.4484
Seroma	69 (5.24%)	153 (5.32%)	0.9176
Infected seroma	4 (0.30%)	12 (0.42%)	0.5808
Hematoma	22 (1.67%)	37 (1.29%)	0.3259
Infected hematoma	1 (0.08%)	7 (0.24%)	0.4484
Exposed biologic mesh	0 (0.00%)	0 (0.00%)	1
Exposed synthetic mesh	5 (0.38%)	4 (0.14%)	0.1501
Contaminated biologic mesh	0 (0.00%)	0 (0.00%)	1
Contaminated synthetic mesh	3 (0.23%)	1 (0.03%)	0.0945
Infected biologic mesh	0 (0.00%)	0 (0.00%)	1
Infected synthetic mesh	2 (0.15%)	3 (0.10%)	0.6522
Mucocutaneous anastomosis disruption	0 (0.00%)	0 (0.00%)	1
Enterocutaneous fistula	1 (0.08%)	1 (0.03%)	0.5294
Unspecified surgical site occurrence	8 (0.61%)	3 (0.10%)	0.0058
Superficial SSI	68 (5.16%)	109 (3.79%)	0.0397
Deep incisional SSI	43 (3.26%)	39 (1.36%)	0
Organ space SSI	4 (0.30%)	2 (0.07%)	0.0819

Categorical variables presented as *n* (%). Categorical tests via Chi-square or Fisher’s exact test if counts ≤ 5

### Multivariable analyses

**Fig. 1:**
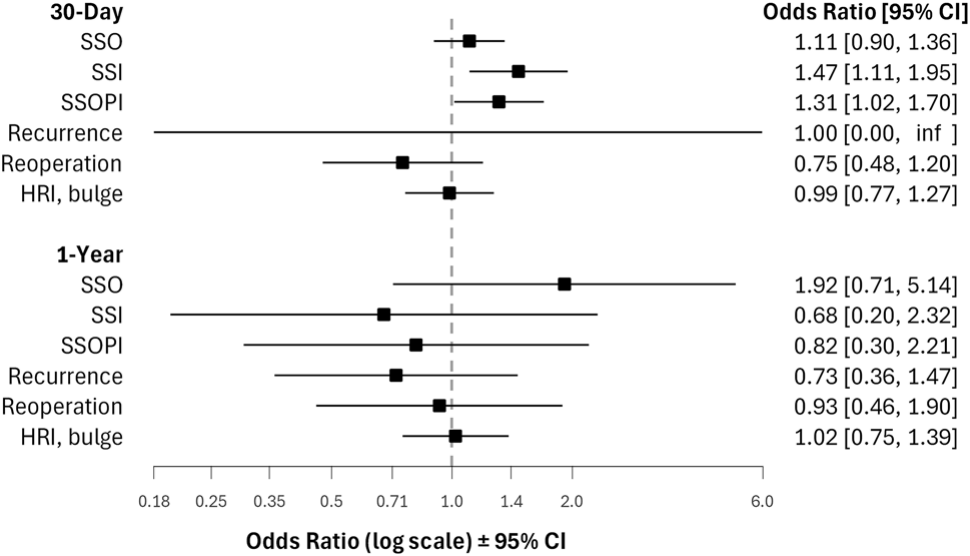
Forest plot illustrating odds ratios (and 95% confidence intervals) of 30-day and -1-year operative outcomes for figure-of-eight vs running suture fascial closure after adjusting for covariates via multivariable logistic regression. Upper confidence interval for 30-day recurrence is infinite due to low event rate (*n* = 2). *SSO* surgical site occurrence, *SSI* surgical site infection, *SSO/I-PI* surgical site occurrence/infections requiring procedural intervention, *HRI* hernia recurrence inventory, *CI* confidence interval, inf infinite

Our primary analysis found no difference in risk of 30-day SSO between figure-of-eight and running suture closure (OR 1.11, 95% CI 0.90, 1.36) after adjusting for patient and surgical factors (Fig. 1). Similarly, no difference in 1-year recurrence odds was identified (OR 0.73, 95% CI 0.36, 1.47). Following multivariable regression, the associations between figure-of-eight closure and 30-day SSI and 30-day SSO/I-PI were redemonstrated. Odds of 30-day SSI were increased by 47% for the figure-of-eight cohort (OR 1.47, 95% CI 1.11, 1.95) and odds of SSO/I-PI were increased by 31% (OR 1.31, 95% CI 1.02, 1.70). The remaining 30-day outcomes remained unassociated with either fascial closure method following adjustment, and no 1-year surgical outcomes were associated with closure technique (Fig. 1).

**Fig. 2:**
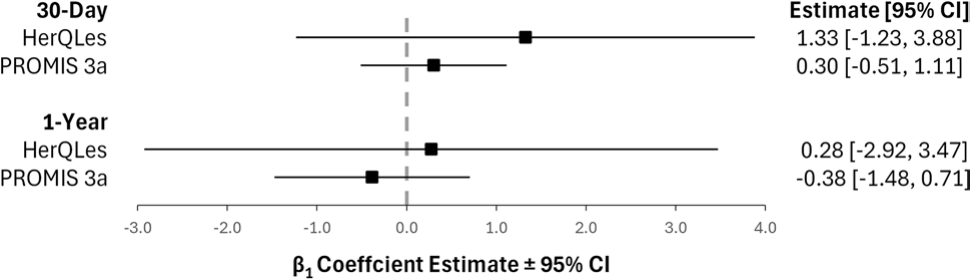
Forest plot illustrating the B coefficient (and 95% confidence intervals) of 30-day and 1-year patient-reported outcomes for figure-of-eight vs running suture fascial closure after adjusting for covariates via multivariable linear regression. *HerQLes* hernia-related quality-of-life survey score, *PROMIS 3a* NIH PROMIS Pain Severity Scale short form survey, *CI* confidence interval

No difference in hernia-related quality of life or pain scores was identified at either timepoint following multivariable linear regression (Fig. 2). Full regression results are available in the Supplemental Document.

## Discussion

This analysis of 4195 open repairs of ≥ 10 cm ventral hernias within a prospectively maintained hernia-specific outcomes registry identified preliminary associations between figure-of-eight fascial closure and wound morbidity vs running suture closure. Though the prespecified primary surrogate of fascial suture line ischemia, 30-day SSO, was not associated with figure-of-eight closure in unadjusted or multivariable analyses, higher odds of 30-day SSI (OR 1.47, 95% CI 1.11, 1.95) and SSO/I-PI (OR 1.31, 95% CI 1.02, 1.70) were observed for figure-of-eights when compared to running suture closures. Importantly, no differences in recurrence or reoperation were demonstrated regarding either closure method; nor were differences in patient-reported outcomes (at either time point) or any clinical outcome at 1-year.

Regarding the primary aim of assessing clinically pertinent ischemia, we hypothesized that 30-day SSO represented a pragmatic surrogate for clinically significant ischemia. Multiple suture line failure outcomes/events are categorized as SSOs within the ACHQC, including fascial disruption, skin/soft tissue ischemia, skin/soft tissue necrosis, and exposed mesh, among others (Supplement 1, Table 1). While suture line ischemia is not the only possible mechanism for such outcomes, our methodology attempted to control for other known risks via eligibility criteria and multivariable regression. Within the confines of the prespecified primary outcome, the results initially appeared suggestive against an ‘ischemic’ quality or association of figure-of-eight fascial closure, but this is predicated upon the validity of SSO as an appropriate surrogate outcome. Tissue necrosis and devitalization can present as SSIs and an increased SSI rate could be attributed to inadequate perfusion. Thus, our secondary findings contribute early data to surprisingly underdeveloped literature, and the observations of increased 30-day SSI and SSO/I-PI odds contrast prior studies suggesting non-difference between figure-of-eight and running suture closure.

Limited literature directly considers figure-of-eight vs running suture fascial closure regarding perfusion or ischemia. Only one study reports differential perfusion patterns for figure-of-eight vs continuous running closures, though for midline laparotomy closure in an animal model rather than hernia repair in human subjects [19]. Using a porcine model, suture line perfusion was assed via indocyanine green laser-induced fluorescence angiography (ICG-FA) intensity at case start, laparotomy closure, and one week; inflammation, neovascularity, and necrosis were also measured at one week. While the relative increase in perfusion at one week was greater for the small bite (5 mm) running suture 0-Polydioxanone (PDS) group vs figure-of-eight 0-PDS group, no differences in immediate postoperative perfusion reduction (vs preoperative), neovascularity at one week, nor necrosis at one week were observed between these groups. Rather, small bite running suture PDS closure was associated with greater suture-entry inflammation and total inflammation vs figure-of-eight closure. The relevance of these results to human VHR is unclear and confounded by sample size, short timeframe, lack of standardization or measurement of fascial closure pressure/tension, and use of a primary laparotomy model (as opposed to an incisional hernia model) but the data appear to not suggest meaningful vascular compromise for figure-of-eights as opposed to running closure.

Clinical outcomes data for figure-of-eights vs running sutures specifically regarding herniorrhaphy are similarly scarce. Only two studies, both utilizing the ACHQC registry, were identified in our literature review. Fox et al. conducted a registry-wide analysis of 6544 cases from 2013 to 2017 to characterize a variety of fascial closure characteristics (i.e., technique, suture type, etc.) [3]. Though inclusion criteria, methodology, and case volume differed from our study, they identified no increased risk of SSO or SSI for figure-of-eight closure vs running suture closure (at 30 days and 6 months). More recently, Remulla et al. demonstrated an association between figure-of-eight utilization and closure of wider hernias at our center, specifically those ≥ 15 cm [7]. This not only demonstrated the utility of figure-of-eight in accomplishing difficult fascial closure but also suggested utilization of figure-of-eight is likely a surrogate of hernia complexity in regard to the degree of fascial tension required for closure and perhaps the quality of the tissue being closed (neither of which could be accounted for in this analysis).

Confounding by indication is an essential consideration when interpreting our results and likely contributed to the increased 30-day SSI and SSO/I-PI rates within the figure-of-eight cohort—perhaps accounting for the observed differences altogether. At our center, some surgeons routinely use figure-of-eight for all hernias requiring component separation, while others selectively utilize figure-of-eights only when running closure could not achieve satisfactory reapproximation. Other centers similarly report selective implementation of interrupted closure techniques (particularly, figure-of-eights) when continuous running suture alone is insufficient; thus, in a hernia-specific registry, running suture closure for large hernias is likely a surrogate for decreased closure tension/complexity [20, 21]. This hypothesis is supported by observation of larger hernia sizes in the figure-of-eights group and differential rates of transversus abdominis release (92% for figure-of-eight closures vs 72% for running suture closures, *P* < 0.001) and Rives-Stoppa utilization (4% for figure-of-eight closures vs 16% for running suture closure, *P* < 0.001), all of which suggest higher complexity in the figure-of-eight cohort, although we could not adequately adjust for specific release type/extent in our analysis. The regression already excluded bridging repairs and accounted for hernia width, hernia length, and the presence of (any) myofascial release; additional adjustment for specific releases would risk overadjustment while still not adequately controlling tissue quality or tension.

Even if tension could be accounted for in this analysis, the clinical significance of increased fascial closure tension in the setting of complex hernia repair (particularly for cases not amenable to running suture closure) is incompletely understood at-large. A prior study of closure tension for large ventral hernia repairs found no association with SSO or SSI at 30-days; however, the study was limited to a cohort featuring a median hernia width of 13 cm (IQR 10.0, 15.2) and mean closure tension of 6.24 lbs, only ~ 4 lbs greater than that of primary laparotomies (and while figure-of-eight and running sutures were both allowed, utilization rates were not reported) [22, 23]. In contrast, the figure-of-eight arm of the present study featured larger hernias (median hernia width = 15 cm (IQR 13, 20)). It is possible tension-SSI association(s) do exist, but only in particularly large or complex ventral hernia repair contexts—situations in which figure-of-eights are more likely to be obligatory, rather than preferential. This conjecture, however, is purely hypothetical and targeted investigation would be required to substantiate any such claim.

To that end, it would be difficult to power or design a prospective study investigating the apparent association of wound morbidity for figure-of-eights (vs running closures). If the wound morbidity association is driven by especially complex or large hernias, the low quantity of such cases would render the study difficult to complete, and it would be unethical to randomize participants to receive running suture if doing so precluded fascial closure. Therefore, inferences drawn from prospectively maintained, hernia-specific registries offer the best available insight at present (and perhaps ever). It is important to note, however, that prospective studies are not necessarily required to inform reasonable practice patterns. Our results position running suture closure as an easy default technique with possible benefit and minimal downside— assuming primary fascial closure is achieved—and multiple studies have demonstrated increased operative efficiency for continuous rather than interrupted fascial closure [4, 24].

Conversely, even if the risks of 30-day infection were redemonstrated in higher fidelity investigations (somehow controlling for tissue quality and/or tension), the observed wound morbidity association would not negate the utility of figure-of-eight closure for fascial defects refractory to running suture closure. The goals of hernioplasty are reduction of sac contents and durable closure of fascia. The consequences of non-fascial closure are well described, with multiple studies demonstrating higher risk of recurrence, higher risk of SSO, and worse patient QoL when bridging is done [25–28]. The present study compared figure-of-eights vs running suture only in cases where closure was achieved; and despite a possibly inflated (and unadjusted) complexity for figure-of-eight cases, recurrence at 1-year was no different vs running suture, suggesting figure-of-eights are effective for durable fascial closure even in complex repairs. Whenever running suture cannot close fascia, the present data are irrelevant—in such circumstances, the risk/benefit consideration would weigh a possible increase in SSI via figure-of-eights vs substantially increased risk of morbidity, recurrence, and diminished quality of life known to accompany bridging/non-closure.

### Limitations

The manuscript holds several key limitations which should be acknowledged. Many are inherent to retrospective registry-based research. First, by leveraging an existing surgical registry, the present methodology could only indirectly test our hypothesis that figure-of-eight midline fascial closure is not associated with suture line ischemia. Second, our design was limited to presently available variables in the ACQHC. The ACHQC does not track dimensional data regarding figure-of-eight or running suture techniques (i.e., 5 mm vs 10 mm bites), needle size, suture size, nor suture material (other than absorbable vs permanent), all of which would be important factors to control for in a prospectively designed comparison between suture techniques. As previously mentioned, we also cannot ascertain why surgeons utilized figure-of-eights or running suture in any situation, differing practices and thresholds for technique implementation introduce substantial heterogeneity. Further, while the study design controlled known confounders via case selection and multivariable analysis, residual confounders may have influenced the results. A final point of consideration is the attrition rate between 30-day follow-up and 1-year follow-up (38%). The primary hypotheses regarded early outcomes and selected for patients as-such; while a sufficient number of patients (*n* = 414 figure-of-eight closures and *n* = 755 running suture closures with complete 1-year follow-up) remained in each cohort to repeat the multivariable regressions at 1-year, the groups were not necessarily adequately powered to detect differences due to the nature of binary, low-incidence outcomes. Therefore, the 1-year outcomes deserve reinvestigation within populations featuring improved long-term data capture.

## Conclusion

Limitations aside, this retrospective analysis demonstrated potential associations between figure-of-eight closure and wound morbidity, suggesting running suture as the first-line fascial closure technique when possible. The previously described benefits of figure-of-eight closure for poor quality fascia, high-tension reapproximation, and/or wide-defect repair are not disputed. Our results reaffirm figure-of-eight closure as safe and useful, but imply the technique should be reserved for high-tension closures to avoid bridging repairs.

## Supplementary Information

Below is the link to the electronic supplementary material.

Supplementary file1 (DOCX 315 kb)
