# Supplementary material for: Figure-of-eights vs running suture for fascial closure of large ventral hernias: do figure-of-eights induce ischemia?
Source: Surg Endosc. 2026 Jun 30;40(8):7001–9. doi: 10.1007/s00464-026-13036-5 (PMC13457274; doi:10.1007/s00464-026-13036-5)
Supplement: Supplementary file 1 [file 464_2026_13036_MOESM1_ESM.docx]

**Supplemental Document 1**

Artificial Intelligence Use Disclosure Statement

**Supplemental Figure 1.** STROBE Flow Diagram of Participant Inclusion

**Supplemental Table 1.** Eligible events within the registry for surgical site occurrences.

**Supplemental Table 2.** Surgical Site Occurrences (SSO) at 30 Days

**Supplemental Table 3**. Surgical Site Occurrences (SSO) at 1 Year

**Supplemental Table 4**. Surgical Site Infections (SSI) at 30 Days

**Supplemental Table 5.** Surgical Site Infections (SSI) at 1 Year

**Supplemental Table 6.** Surgical Site Occurrences/Infections Requiring Procedural Intervention (SSO/I-PI) at 30 Days

**Supplemental Table 7.** Surgical Site Occurrences/Infections Requiring Procedural Intervention (SSO/I-PI) at 1 Year

**Supplemental Table 8.** Recurrence at 30 Days

**Supplemental Table 9.** Recurrence at 1 Year

**Supplemental Table 10.** Reoperation at 30 Days

**Supplemental Table 11.** Reoperation at 1 Year

**Supplemental Table 12.** HerQLes Score at 30 Days

**Supplemental Table 13.** HerQLes Score at 1 Year

**Supplemental Table 14.** PROMIS Pain 3a T-Score at 30 Days

**Supplemental Table 15**. PROMIS Pain 3a T-Score at 1 Year

**Supplemental Table 16.** HRI: Patient-Reported Recurrence (Bulge) at 30 Days

**Supplemental Table 17.** HRI: Patient-Reported Recurrence (Bulge) at 1 Year

**Artificial Intelligence Use Disclosure Statement:**

An artificial intelligence (AI) model (Claude Opus 4.7, 4/30/2026; Anthropic) was utilized to convert unformatted tables of linear and logistic regression results (analysis already conducted by statistician) into tables formatted for .docx, specifically generating Supplemental Tables 2 – 17 to comply with publisher file type requirements for manuscript components. Tables were checked and confirmed for accuracy. AI was not otherwise used for any part of the research project including study design, data collection, statistical analysis, manuscript drafting, manuscript review, or manuscript submission.

**Supplemental Figure 1.** STROBE Flow Diagram of Participant Inclusion

**Supplemental Table 1.** Eligible events within the registry for surgical site occurrences.

ACHQC Surgical Site Occurrences

Wound Cellulitis

Non-healing incisional wound

Fascial disruption

Skin or soft tissue ischemia

skin or soft tissue necrosis

Wound serous drainage

Wound purulent drainage

Chronic sinus drainage

Localized stab wound infection

Stitch abscess

Seroma

Infected Seroma

Hematoma

Infected hematoma

Exposed biological mesh

Exposed synthetic mesh

Contaminated synthetic mesh

Infected biologic mesh

Infected synthetic mesh

Mucocutaneous anastomosis disruption

Enterocutaneous fistula

Unspecified surgical site occurrence

**Supplemental Table 2.** Surgical Site Occurrences (SSO) at 30 Days

| **Variable** | **Contrast** | **Low** | **High** | **Δ** | **β** | **SE** | **95% CI Lower** | **95% CI Upper** | **Odds Ratio (95% CI)** |
| --- | --- | --- | --- | --- | --- | --- | --- | --- | --- |
| Hernia length (cm) |  | 15 | 25 | 10 | 0.009 | 0.084 | -0.156 | 0.174 | **1.01 (0.86–1.19)** |
| Hernia width (cm) |  | 12 | 17 | 5 | 0.074 | 0.050 | -0.024 | 0.172 | **1.08 (0.98–1.19)** |
| BMI |  | 29 | 36.8 | 7.8 | 0.289 | 0.057 | 0.178 | 0.401 | **1.34 (1.20–1.49)** |
| Age |  | 51 | 68 | 17 | -0.128 | 0.070 | -0.266 | 0.010 | **0.88 (0.77–1.01)** |
| **Fascial Closure Technique** | **Fo8 vs Running** | 2 | 1 | — | 0.102 | 0.104 | -0.102 | 0.306 | **1.11 (0.90–1.36)** |
| Procedure: Epigastric (primary ventral) | Yes vs No | 2 | 1 | — | 0.142 | 0.843 | -1.511 | 1.795 | **1.15 (0.22–6.02)** |
| Procedure: Incisional | No vs Yes | 1 | 2 | — | -1.257 | 0.580 | -2.393 | -0.121 | **0.28 (0.09–0.89)** |
| Recurrent | No vs Yes | 1 | 2 | — | -0.230 | 0.095 | -0.416 | -0.045 | **0.79 (0.66–0.96)** |
| Gender | Male vs Female | 2 | 1 | — | -0.065 | 0.094 | -0.249 | 0.119 | **0.94 (0.78–1.13)** |
| Race: American Indian / Alaskan Native | vs White | 5 | 1 | — | -0.750 | 1.055 | -2.818 | 1.317 | **0.47 (0.06–3.73)** |
| Race: Asian, Native Hawaiian / Other Pacific | vs White | 5 | 2 | — | -0.308 | 1.061 | -2.387 | 1.772 | **0.73 (0.09–5.88)** |
| Race: Black or African American | vs White | 5 | 3 | — | 0.115 | 0.186 | -0.251 | 0.481 | **1.12 (0.78–1.62)** |
| Race: Hispanic | vs White | 5 | 4 | — | 0.208 | 0.271 | -0.323 | 0.740 | **1.23 (0.72–2.10)** |
| Race: Middle Eastern | vs White | 5 | 6 | — | — | 326.700 | — | — | **Not estimable** |
| Race: Other or unknown | vs White | 5 | 7 | — | -0.443 | 0.620 | -1.658 | 0.772 | **0.64 (0.19–2.16)** |
| Race: Not Indicated | vs White | 5 | 8 | — | -1.027 | 1.030 | -3.045 | 0.992 | **0.36 (0.05–2.70)** |
| ASA Class: 1 | vs 3 | 3 | 1 | — | -0.178 | 0.542 | -1.241 | 0.884 | **0.84 (0.29–2.42)** |
| ASA Class: 2 | vs 3 | 3 | 2 | — | -0.256 | 0.122 | -0.495 | -0.016 | **0.78 (0.61–0.98)** |
| ASA Class: 4 | vs 3 | 3 | 4 | — | 0.185 | 0.255 | -0.315 | 0.685 | **1.20 (0.73–1.98)** |
| ASA Class: None Assigned | vs 3 | 3 | 5 | — | — | 706.200 | — | — | **Not estimable** |
| Immunosuppressants | Yes vs No | 2 | 1 | — | 0.174 | 0.164 | -0.147 | 0.495 | **1.19 (0.86–1.64)** |
| Smoking (current within 1 mo) | Yes vs No | 2 | 1 | — | 0.504 | 0.158 | 0.194 | 0.814 | **1.66 (1.21–2.26)** |
| Diabetes Mellitus | Yes vs No | 2 | 1 | — | 0.164 | 0.105 | -0.042 | 0.370 | **1.18 (0.96–1.45)** |
| Elective Case | No vs Yes | 1 | 2 | — | -0.122 | 0.335 | -0.778 | 0.533 | **0.89 (0.46–1.71)** |
| Myofascial Release | No vs Yes | 1 | 2 | — | 0.423 | 0.154 | 0.121 | 0.725 | **1.53 (1.13–2.07)** |
| Drains used | No vs Yes | 1 | 2 | — | 0.279 | 0.220 | -0.152 | 0.709 | **1.32 (0.86–2.03)** |
| Concomitant procedure | Yes vs No | 2 | 1 | — | 0.141 | 0.124 | -0.103 | 0.384 | **1.15 (0.90–1.47)** |

**Summary:** Odds of a 30-day surgical site occurrence are increased by 11% for Fo8 vs running suture; not statistically significant (95% CI: 0.90–1.36), adjusting for patient and surgical characteristics.

Δ = magnitude of contrast (High − Low) for continuous variables. β = log-odds coefficient. OR = adjusted odds ratio with 95% CI. "Not estimable" indicates convergence failure (typically due to zero/empty cells). Yellow-highlighted row = primary comparison (Fo8 vs Running suture).

**Supplemental Table 3.** Surgical Site Occurrences (SSO) at 1 Year

| **Variable** | **Contrast** | **Low** | **High** | **Δ** | **β** | **SE** | **95% CI Lower** | **95% CI Upper** | **Odds Ratio (95% CI)** |
| --- | --- | --- | --- | --- | --- | --- | --- | --- | --- |
| Hernia length (cm) |  | 16 | 25 | 9 | 0.212 | 0.387 | -0.547 | 0.972 | **1.24 (0.58–2.64)** |
| Hernia width (cm) |  | 12 | 17 | 5 | 0.204 | 0.205 | -0.198 | 0.606 | **1.23 (0.82–1.83)** |
| BMI |  | 28.7 | 36.4 | 7.72 | 0.383 | 0.309 | -0.224 | 0.988 | **1.47 (0.80–2.69)** |
| Age |  | 53 | 68 | 15 | 0.465 | 0.355 | -0.230 | 1.160 | **1.59 (0.80–3.19)** |
| **Fascial Closure Technique** | **Fo8 vs Running** | 2 | 1 | — | 0.650 | 0.504 | -0.337 | 1.637 | **1.92 (0.71–5.14)** |
| Procedure: Epigastric (primary ventral) | Yes vs No | 2 | 1 | — | — | 2777.000 | — | — | **Not estimable** |
| Procedure: Incisional | No vs Yes | 1 | 2 | — | — | 2014.000 | — | — | **Not estimable** |
| Recurrent | No vs Yes | 1 | 2 | — | -0.608 | 0.490 | -1.568 | 0.351 | **0.54 (0.21–1.42)** |
| Gender | Male vs Female | 2 | 1 | — | -0.307 | 0.479 | -1.246 | 0.630 | **0.73 (0.29–1.88)** |
| Race: American Indian / Alaskan Native | vs White | 5 | 1 | — | — | 3875.000 | — | — | **Not estimable** |
| Race: Asian, Native Hawaiian / Other Pacific | vs White | 5 | 2 | — | — | 4994.000 | — | — | **Not estimable** |
| Race: Black or African American | vs White | 5 | 3 | — | 1.196 | 0.723 | -0.222 | 2.614 | **3.31 (0.80–13.65)** |
| Race: Hispanic | vs White | 5 | 4 | — | — | 1081.000 | — | — | **Not estimable** |
| Race: Middle Eastern | vs White | 5 | 6 | — | — | 1581.000 | — | — | **Not estimable** |
| Race: Other or unknown | vs White | 5 | 7 | — | — | 2072.000 | — | — | **Not estimable** |
| Race: Not Indicated | vs White | 5 | 8 | — | — | 2569.000 | — | — | **Not estimable** |
| ASA Class: 1 | vs 3 | 3 | 1 | — | — | 2089.000 | — | — | **Not estimable** |
| ASA Class: 2 | vs 3 | 3 | 2 | — | -0.526 | 0.778 | -2.051 | 0.999 | **0.59 (0.13–2.72)** |
| ASA Class: 4 | vs 3 | 3 | 4 | — | 0.232 | 0.861 | -1.455 | 1.919 | **1.26 (0.23–6.81)** |
| Immunosuppressants | Yes vs No | 2 | 1 | — | 0.846 | 0.650 | -0.427 | 2.119 | **2.33 (0.65–8.33)** |
| Smoking (current within 1 mo) | Yes vs No | 2 | 1 | — | 1.287 | 0.836 | -0.351 | 2.926 | **3.62 (0.70–18.64)** |
| Diabetes Mellitus | Yes vs No | 2 | 1 | — | 0.706 | 0.468 | -0.210 | 1.623 | **2.03 (0.81–5.07)** |
| Elective Case | No vs Yes | 1 | 2 | — | — | 984.300 | — | — | **Not estimable** |
| Myofascial Release | No vs Yes | 1 | 2 | — | 0.912 | 0.771 | -0.600 | 2.423 | **2.49 (0.55–11.28)** |
| Drains used | No vs Yes | 1 | 2 | — | 0.990 | 1.148 | -1.260 | 3.239 | **2.69 (0.28–25.50)** |
| Concomitant procedure | Yes vs No | 2 | 1 | — | 1.015 | 0.568 | -0.098 | 2.128 | **2.76 (0.91–8.40)** |

**Summary:** Odds of a 1-year surgical site occurrence are increased by 92% for Fo8 vs running suture; not statistically significant (95% CI: 0.71–5.14), adjusting for patient and surgical characteristics.

Δ = magnitude of contrast (High − Low) for continuous variables. β = log-odds coefficient. OR = adjusted odds ratio with 95% CI. "Not estimable" indicates convergence failure (typically due to zero/empty cells). Yellow-highlighted row = primary comparison (Fo8 vs Running suture).

**Supplemental Table 4.** Surgical Site Infections (SSI) at 30 Days

| **Variable** | **Contrast** | **Low** | **High** | **Δ** | **β** | **SE** | **95% CI Lower** | **95% CI Upper** | **Odds Ratio (95% CI)** |
| --- | --- | --- | --- | --- | --- | --- | --- | --- | --- |
| Hernia length (cm) |  | 15 | 25 | 10 | 0.171 | 0.126 | -0.075 | 0.417 | **1.19 (0.93–1.52)** |
| Hernia width (cm) |  | 12 | 17 | 5 | 0.044 | 0.074 | -0.100 | 0.189 | **1.04 (0.91–1.21)** |
| BMI |  | 29 | 36.8 | 7.8 | 0.304 | 0.079 | 0.148 | 0.460 | **1.35 (1.16–1.58)** |
| Age |  | 51 | 68 | 17 | -0.137 | 0.102 | -0.337 | 0.063 | **0.87 (0.71–1.06)** |
| **Fascial Closure Technique** | **Fo8 vs Running** | 2 | 1 | — | 0.385 | 0.144 | 0.102 | 0.667 | **1.47 (1.11–1.95)** |
| Procedure: Epigastric (primary ventral) | Yes vs No | 2 | 1 | — | — | 462.800 | — | — | **Not estimable** |
| Procedure: Incisional | No vs Yes | 1 | 2 | — | 0.234 | 0.611 | -0.963 | 1.430 | **1.26 (0.38–4.18)** |
| Recurrent | No vs Yes | 1 | 2 | — | -0.406 | 0.140 | -0.680 | -0.132 | **0.67 (0.51–0.88)** |
| Gender | Male vs Female | 2 | 1 | — | -0.271 | 0.136 | -0.537 | -0.004 | **0.76 (0.58–1.00)** |
| Race: American Indian / Alaskan Native | vs White | 5 | 1 | — | 0.215 | 1.058 | -1.860 | 2.289 | **1.24 (0.16–9.86)** |
| Race: Asian, Native Hawaiian / Other Pacific | vs White | 5 | 2 | — | — | 763.900 | — | — | **Not estimable** |
| Race: Black or African American | vs White | 5 | 3 | — | -0.188 | 0.300 | -0.775 | 0.400 | **0.83 (0.46–1.49)** |
| Race: Hispanic | vs White | 5 | 4 | — | 0.044 | 0.410 | -0.759 | 0.847 | **1.04 (0.47–2.33)** |
| Race: Middle Eastern | vs White | 5 | 6 | — | -0.517 | 1.025 | -2.526 | 1.493 | **0.60 (0.08–4.45)** |
| Race: Other or unknown | vs White | 5 | 7 | — | -0.643 | 1.024 | -2.651 | 1.364 | **0.53 (0.07–3.91)** |
| Race: Not Indicated | vs White | 5 | 8 | — | 0.064 | 1.035 | -1.964 | 2.092 | **1.07 (0.14–8.10)** |
| ASA Class: 1 | vs 3 | 3 | 1 | — | -0.479 | 1.028 | -2.494 | 1.535 | **0.62 (0.08–4.64)** |
| ASA Class: 2 | vs 3 | 3 | 2 | — | -0.092 | 0.176 | -0.437 | 0.252 | **0.91 (0.65–1.29)** |
| ASA Class: 4 | vs 3 | 3 | 4 | — | 0.419 | 0.339 | -0.245 | 1.083 | **1.52 (0.78–2.96)** |
| ASA Class: None Assigned | vs 3 | 3 | 5 | — | — | 1091.000 | — | — | **Not estimable** |
| Immunosuppressants | Yes vs No | 2 | 1 | — | -0.197 | 0.267 | -0.720 | 0.326 | **0.82 (0.49–1.39)** |
| Smoking (current within 1 mo) | Yes vs No | 2 | 1 | — | 0.271 | 0.239 | -0.197 | 0.740 | **1.31 (0.82–2.10)** |
| Diabetes Mellitus | Yes vs No | 2 | 1 | — | 0.148 | 0.149 | -0.144 | 0.440 | **1.16 (0.87–1.55)** |
| Elective Case | No vs Yes | 1 | 2 | — | 0.723 | 0.378 | -0.018 | 1.464 | **2.06 (0.98–4.33)** |
| Myofascial Release | No vs Yes | 1 | 2 | — | -0.065 | 0.261 | -0.575 | 0.446 | **0.94 (0.56–1.56)** |
| Drains used | No vs Yes | 1 | 2 | — | -0.390 | 0.435 | -1.243 | 0.463 | **0.68 (0.29–1.59)** |
| Concomitant procedure | Yes vs No | 2 | 1 | — | -0.108 | 0.192 | -0.485 | 0.268 | **0.90 (0.62–1.31)** |

**Summary:** Odds of a 30-day surgical site infection are increased by 47% for Fo8 vs running suture. Statistically significant (95% CI: 1.11–1.95), adjusting for patient and surgical characteristics.

Δ = magnitude of contrast (High − Low) for continuous variables. β = log-odds coefficient. OR = adjusted odds ratio with 95% CI. "Not estimable" indicates convergence failure (typically due to zero/empty cells). Yellow-highlighted row = primary comparison (Fo8 vs Running suture).

**Supplemental Table 5.** Surgical Site Infections (SSI) at 1 Year

| **Variable** | **Contrast** | **Low** | **High** | **Δ** | **β** | **SE** | **95% CI Lower** | **95% CI Upper** | **Odds Ratio (95% CI)** |
| --- | --- | --- | --- | --- | --- | --- | --- | --- | --- |
| Hernia length (cm) |  | 16 | 25 | 9 | 0.410 | 0.551 | -0.670 | 1.490 | **1.51 (0.51–4.44)** |
| Hernia width (cm) |  | 12 | 17 | 5 | -0.108 | 0.315 | -0.726 | 0.509 | **0.90 (0.48–1.66)** |
| BMI |  | 28.7 | 36.4 | 7.72 | 0.034 | 0.399 | -0.748 | 0.817 | **1.03 (0.47–2.26)** |
| Age |  | 53 | 68 | 15 | 0.111 | 0.434 | -0.740 | 0.962 | **1.12 (0.48–2.62)** |
| **Fascial Closure Technique** | **Fo8 vs Running** | 2 | 1 | — | -0.390 | 0.628 | -1.621 | 0.841 | **0.68 (0.20–2.32)** |
| Procedure: Epigastric (primary ventral) | Yes vs No | 2 | 1 | — | — | 3987.000 | — | — | **Not estimable** |
| Procedure: Incisional | No vs Yes | 1 | 2 | — | — | 3085.000 | — | — | **Not estimable** |
| Recurrent | No vs Yes | 1 | 2 | — | -1.085 | 0.682 | -2.422 | 0.252 | **0.34 (0.09–1.29)** |
| Gender | Male vs Female | 2 | 1 | — | -0.058 | 0.585 | -1.205 | 1.089 | **0.94 (0.30–2.97)** |
| Race: American Indian / Alaskan Native | vs White | 5 | 1 | — | — | 7166.000 | — | — | **Not estimable** |
| Race: Asian, Native Hawaiian / Other Pacific | vs White | 5 | 2 | — | — | 9298.000 | — | — | **Not estimable** |
| Race: Black or African American | vs White | 5 | 3 | — | — | 1324.000 | — | — | **Not estimable** |
| Race: Hispanic | vs White | 5 | 4 | — | — | 2493.000 | — | — | **Not estimable** |
| Race: Middle Eastern | vs White | 5 | 6 | — | — | 3506.000 | — | — | **Not estimable** |
| Race: Other or unknown | vs White | 5 | 7 | — | — | 4208.000 | — | — | **Not estimable** |
| Race: Not Indicated | vs White | 5 | 8 | — | — | 5010.000 | — | — | **Not estimable** |
| ASA Class: 1 | vs 3 | 3 | 1 | — | — | 3918.000 | — | — | **Not estimable** |
| ASA Class: 2 | vs 3 | 3 | 2 | — | -1.032 | 1.081 | -3.151 | 1.088 | **0.36 (0.04–2.97)** |
| ASA Class: 4 | vs 3 | 3 | 4 | — | 1.710 | 0.912 | -0.077 | 3.497 | **5.53 (0.93–33.00)** |
| Immunosuppressants | Yes vs No | 2 | 1 | — | 0.181 | 0.882 | -1.547 | 1.909 | **1.20 (0.21–6.75)** |
| Smoking (current within 1 mo) | Yes vs No | 2 | 1 | — | 0.662 | 1.114 | -1.522 | 2.846 | **1.94 (0.22–17.22)** |
| Diabetes Mellitus | Yes vs No | 2 | 1 | — | 0.035 | 0.640 | -1.219 | 1.289 | **1.04 (0.30–3.63)** |
| Elective Case | No vs Yes | 1 | 2 | — | — | 2416.000 | — | — | **Not estimable** |
| Myofascial Release | No vs Yes | 1 | 2 | — | — | 1120.000 | — | — | **Not estimable** |
| Drains used | No vs Yes | 1 | 2 | — | — | 1907.000 | — | — | **Not estimable** |
| Concomitant procedure | Yes vs No | 2 | 1 | — | -0.152 | 1.069 | -2.247 | 1.943 | **0.86 (0.11–6.98)** |

**Summary:** Odds of a 1-year surgical site infection are decreased by 32% for Fo8 vs running suture; not statistically significant (95% CI: 0.20–2.32), adjusting for patient and surgical characteristics.

Δ = magnitude of contrast (High − Low) for continuous variables. β = log-odds coefficient. OR = adjusted odds ratio with 95% CI. "Not estimable" indicates convergence failure (typically due to zero/empty cells). Yellow-highlighted row = primary comparison (Fo8 vs Running suture).

**Supplemental Table 6.** Surgical Site Occurrences/Infections Requiring Procedural Intervention (SSO/I-PI) at 30 Days

| **Variable** | **Contrast** | **Low** | **High** | **Δ** | **β** | **SE** | **95% CI Lower** | **95% CI Upper** | **Odds Ratio (95% CI)** |
| --- | --- | --- | --- | --- | --- | --- | --- | --- | --- |
| Hernia length (cm) |  | 15 | 25 | 10 | 0.183 | 0.113 | -0.038 | 0.405 | **1.20 (0.96–1.50)** |
| Hernia width (cm) |  | 12 | 17 | 5 | 0.040 | 0.067 | -0.090 | 0.171 | **1.04 (0.91–1.19)** |
| BMI |  | 29 | 36.8 | 7.8 | 0.331 | 0.072 | 0.191 | 0.472 | **1.39 (1.21–1.60)** |
| Age |  | 51 | 68 | 17 | -0.105 | 0.092 | -0.286 | 0.076 | **0.90 (0.75–1.08)** |
| **Fascial Closure Technique** | **Fo8 vs Running** | 2 | 1 | — | 0.273 | 0.131 | 0.015 | 0.530 | **1.31 (1.01–1.70)** |
| Procedure: Epigastric (primary ventral) | Yes vs No | 2 | 1 | — | — | 654.800 | — | — | **Not estimable** |
| Procedure: Incisional | No vs Yes | 1 | 2 | — | -0.214 | 0.610 | -1.409 | 0.982 | **0.81 (0.24–2.67)** |
| Recurrent | No vs Yes | 1 | 2 | — | -0.276 | 0.124 | -0.520 | -0.032 | **0.76 (0.59–0.97)** |
| Gender | Male vs Female | 2 | 1 | — | -0.230 | 0.123 | -0.470 | 0.011 | **0.80 (0.63–1.01)** |
| Race: American Indian / Alaskan Native | vs White | 5 | 1 | — | — | 1115.000 | — | — | **Not estimable** |
| Race: Asian, Native Hawaiian / Other Pacific | vs White | 5 | 2 | — | — | 1152.000 | — | — | **Not estimable** |
| Race: Black or African American | vs White | 5 | 3 | — | -0.324 | 0.280 | -0.872 | 0.225 | **0.72 (0.42–1.25)** |
| Race: Hispanic | vs White | 5 | 4 | — | 0.060 | 0.370 | -0.665 | 0.785 | **1.06 (0.51–2.19)** |
| Race: Middle Eastern | vs White | 5 | 6 | — | -0.789 | 1.026 | -2.800 | 1.221 | **0.45 (0.06–3.39)** |
| Race: Other or unknown | vs White | 5 | 7 | — | 0.282 | 0.622 | -0.938 | 1.502 | **1.32 (0.39–4.49)** |
| Race: Not Indicated | vs White | 5 | 8 | — | -0.125 | 1.035 | -2.153 | 1.903 | **0.88 (0.12–6.71)** |
| ASA Class: 1 | vs 3 | 3 | 1 | — | — | 594.300 | — | — | **Not estimable** |
| ASA Class: 2 | vs 3 | 3 | 2 | — | -0.466 | 0.172 | -0.804 | -0.128 | **0.63 (0.45–0.88)** |
| ASA Class: 4 | vs 3 | 3 | 4 | — | 0.263 | 0.314 | -0.353 | 0.879 | **1.30 (0.70–2.41)** |
| ASA Class: None Assigned | vs 3 | 3 | 5 | — | — | 1542.000 | — | — | **Not estimable** |
| Immunosuppressants | Yes vs No | 2 | 1 | — | -0.245 | 0.242 | -0.719 | 0.228 | **0.78 (0.49–1.26)** |
| Smoking (current within 1 mo) | Yes vs No | 2 | 1 | — | 0.423 | 0.208 | 0.016 | 0.830 | **1.53 (1.02–2.29)** |
| Diabetes Mellitus | Yes vs No | 2 | 1 | — | 0.290 | 0.131 | 0.034 | 0.546 | **1.34 (1.03–1.73)** |
| Elective Case | No vs Yes | 1 | 2 | — | 0.521 | 0.358 | -0.181 | 1.222 | **1.68 (0.83–3.40)** |
| Myofascial Release | No vs Yes | 1 | 2 | — | 0.326 | 0.213 | -0.092 | 0.744 | **1.39 (0.91–2.10)** |
| Drains used | No vs Yes | 1 | 2 | — | -0.330 | 0.364 | -1.042 | 0.384 | **0.72 (0.35–1.47)** |
| Concomitant procedure | Yes vs No | 2 | 1 | — | -0.009 | 0.168 | -0.338 | 0.321 | **0.99 (0.71–1.38)** |

**Summary:** Odds of a 30-day SSO/I requiring procedural intervention are increased by 31% for Fo8 vs running suture. Statistically significant (95% CI: 1.02–1.70).

Δ = magnitude of contrast (High − Low) for continuous variables. β = log-odds coefficient. OR = adjusted odds ratio with 95% CI. "Not estimable" indicates convergence failure (typically due to zero/empty cells). Yellow-highlighted row = primary comparison (Fo8 vs Running suture).

**Supplemental Table 7.** Surgical Site Occurrences/Infections Requiring Procedural Intervention (SSO/I-PI) at 1 Year

| **Variable** | **Contrast** | **Low** | **High** | **Δ** | **β** | **SE** | **95% CI Lower** | **95% CI Upper** | **Odds Ratio (95% CI)** |
| --- | --- | --- | --- | --- | --- | --- | --- | --- | --- |
| Hernia length (cm) |  | 16 | 25 | 9 | 0.032 | 0.394 | -0.741 | 0.804 | **1.03 (0.48–2.23)** |
| Hernia width (cm) |  | 12 | 17 | 5 | 0.143 | 0.213 | -0.275 | 0.561 | **1.15 (0.76–1.75)** |
| BMI |  | 28.7 | 36.4 | 7.72 | 0.417 | 0.304 | -0.179 | 1.013 | **1.52 (0.84–2.75)** |
| Age |  | 53 | 68 | 15 | 0.244 | 0.336 | -0.414 | 0.903 | **1.28 (0.66–2.47)** |
| **Fascial Closure Technique** | **Fo8 vs Running** | 2 | 1 | — | -0.203 | 0.507 | -1.198 | 0.791 | **0.82 (0.30–2.21)** |
| Procedure: Epigastric (primary ventral) | Yes vs No | 2 | 1 | — | — | 2027.000 | — | — | **Not estimable** |
| Procedure: Incisional | No vs Yes | 1 | 2 | — | — | 1535.000 | — | — | **Not estimable** |
| Recurrent | No vs Yes | 1 | 2 | — | -1.148 | 0.533 | -2.192 | -0.103 | **0.32 (0.11–0.90)** |
| Gender | Male vs Female | 2 | 1 | — | 0.052 | 0.465 | -0.858 | 0.962 | **1.05 (0.42–2.62)** |
| Race: American Indian / Alaskan Native | vs White | 5 | 1 | — | — | 3391.000 | — | — | **Not estimable** |
| Race: Asian, Native Hawaiian / Other Pacific | vs White | 5 | 2 | — | — | 4273.000 | — | — | **Not estimable** |
| Race: Black or African American | vs White | 5 | 3 | — | 1.155 | 0.727 | -0.270 | 2.580 | **3.17 (0.76–13.20)** |
| Race: Hispanic | vs White | 5 | 4 | — | — | 1244.000 | — | — | **Not estimable** |
| Race: Middle Eastern | vs White | 5 | 6 | — | — | 1457.000 | — | — | **Not estimable** |
| Race: Other or unknown | vs White | 5 | 7 | — | — | 1837.000 | — | — | **Not estimable** |
| Race: Not Indicated | vs White | 5 | 8 | — | — | 2262.000 | — | — | **Not estimable** |
| ASA Class: 1 | vs 3 | 3 | 1 | — | — | 1849.000 | — | — | **Not estimable** |
| ASA Class: 2 | vs 3 | 3 | 2 | — | -1.519 | 1.046 | -3.569 | 0.531 | **0.22 (0.03–1.70)** |
| ASA Class: 4 | vs 3 | 3 | 4 | — | 1.038 | 0.740 | -0.412 | 2.489 | **2.83 (0.66–12.05)** |
| Immunosuppressants | Yes vs No | 2 | 1 | — | 0.090 | 0.719 | -1.318 | 1.499 | **1.09 (0.27–4.48)** |
| Smoking (current within 1 mo) | Yes vs No | 2 | 1 | — | 1.153 | 0.831 | -0.476 | 2.782 | **3.17 (0.62–16.15)** |
| Diabetes Mellitus | Yes vs No | 2 | 1 | — | 0.252 | 0.474 | -0.677 | 1.182 | **1.29 (0.51–3.26)** |
| Elective Case | No vs Yes | 1 | 2 | — | — | 1233.000 | — | — | **Not estimable** |
| Myofascial Release | No vs Yes | 1 | 2 | — | -0.043 | 0.884 | -1.777 | 1.690 | **0.96 (0.17–5.42)** |
| Drains used | No vs Yes | 1 | 2 | — | 0.493 | 1.133 | -1.726 | 2.713 | **1.64 (0.18–15.08)** |
| Concomitant procedure | Yes vs No | 2 | 1 | — | 0.577 | 0.607 | -0.613 | 1.767 | **1.78 (0.54–5.85)** |

**Summary:** Odds of a 1-year SSO/I requiring procedural intervention are reduced by 18% for Fo8 vs running suture; not statistically significant (95% CI: 0.30–2.21).

Δ = magnitude of contrast (High − Low) for continuous variables. β = log-odds coefficient. OR = adjusted odds ratio with 95% CI. "Not estimable" indicates convergence failure (typically due to zero/empty cells). Yellow-highlighted row = primary comparison (Fo8 vs Running suture).

**Supplemental Table 8.** Recurrence at 30 Days

| **Variable** | **Contrast** | **Low** | **High** | **Δ** | **β** | **SE** | **95% CI Lower** | **95% CI Upper** | **Odds Ratio (95% CI)** |
| --- | --- | --- | --- | --- | --- | --- | --- | --- | --- |
| Hernia length (cm) |  | 15 | 25 | 10 | — | 880.400 | — | — | **Not estimable** |
| Hernia width (cm) |  | 12 | 17 | 5 | — | 1479.000 | — | — | **Not estimable** |
| BMI |  | 29 | 36.8 | 7.8 | — | 867.200 | — | — | **Not estimable** |
| Age |  | 51 | 68 | 17 | — | 702.300 | — | — | **Not estimable** |
| **Fascial Closure Technique** | **Fo8 vs Running** | 2 | 1 | — | — | 3803.000 | — | — | **Not estimable** |
| All other variables | — | — | — | — | — | — | — | — | **Not estimable** |

**Summary:** With only 2 recurrences observed at 30 days, all confidence intervals are infinite. No reliable inference is possible from this analysis.

***Note:*** *Only 2 30-day recurrences observed. Confidence intervals are infinite/not estimable for all variables; estimates are unreliable.*

Δ = magnitude of contrast (High − Low) for continuous variables. β = log-odds coefficient. OR = adjusted odds ratio with 95% CI. "Not estimable" indicates convergence failure (typically due to zero/empty cells). Yellow-highlighted row = primary comparison (Fo8 vs Running suture).

**Supplemental Table 9.** Recurrence at 1 Year

| **Variable** | **Contrast** | **Low** | **High** | **Δ** | **β** | **SE** | **95% CI Lower** | **95% CI Upper** | **Odds Ratio (95% CI)** |
| --- | --- | --- | --- | --- | --- | --- | --- | --- | --- |
| Hernia length (cm) |  | 16 | 25 | 9 | 0.263 | 0.261 | -0.248 | 0.775 | **1.30 (0.78–2.17)** |
| Hernia width (cm) |  | 12 | 17 | 5 | 0.120 | 0.150 | -0.174 | 0.415 | **1.13 (0.84–1.51)** |
| BMI |  | 28.7 | 36.3 | 7.6 | 0.148 | 0.213 | -0.270 | 0.566 | **1.16 (0.76–1.76)** |
| Age |  | 53 | 68 | 15 | 0.376 | 0.238 | -0.090 | 0.841 | **1.46 (0.91–2.32)** |
| **Fascial Closure Technique** | **Fo8 vs Running** | 2 | 1 | — | -0.320 | 0.358 | -1.021 | 0.382 | **0.73 (0.36–1.47)** |
| Procedure: Epigastric (primary ventral) | Yes vs No | 2 | 1 | — | — | 2037.000 | — | — | **Not estimable** |
| Procedure: Incisional | No vs Yes | 1 | 2 | — | 1.129 | 1.347 | -1.512 | 3.770 | **3.09 (0.22–43.40)** |
| Recurrent | No vs Yes | 1 | 2 | — | -0.306 | 0.330 | -0.952 | 0.341 | **0.74 (0.39–1.41)** |
| Gender | Male vs Female | 2 | 1 | — | -0.121 | 0.325 | -0.758 | 0.516 | **0.89 (0.47–1.68)** |
| Race: American Indian / Alaskan Native | vs White | 5 | 1 | — | — | 2176.000 | — | — | **Not estimable** |
| Race: Asian, Native Hawaiian / Other Pacific | vs White | 5 | 2 | — | — | 2807.000 | — | — | **Not estimable** |
| Race: Black or African American | vs White | 5 | 3 | — | 0.638 | 0.579 | -0.497 | 1.773 | **1.89 (0.61–5.89)** |
| Race: Hispanic | vs White | 5 | 4 | — | — | 859.600 | — | — | **Not estimable** |
| Race: Middle Eastern | vs White | 5 | 6 | — | — | 1421.000 | — | — | **Not estimable** |
| Race: Other or unknown | vs White | 5 | 7 | — | 1.787 | 1.332 | -0.823 | 4.398 | **5.97 (0.44–81.29)** |
| Race: Not Indicated | vs White | 5 | 8 | — | 1.561 | 1.237 | -0.864 | 3.986 | **4.76 (0.42–53.83)** |
| ASA Class: 1 | vs 3 | 3 | 1 | — | — | 1161.000 | — | — | **Not estimable** |
| ASA Class: 2 | vs 3 | 3 | 2 | — | -0.021 | 0.427 | -0.858 | 0.816 | **0.98 (0.42–2.26)** |
| ASA Class: 4 | vs 3 | 3 | 4 | — | -0.984 | 1.077 | -3.094 | 1.126 | **0.37 (0.04–3.08)** |
| Immunosuppressants | Yes vs No | 2 | 1 | — | 1.270 | 0.416 | 0.455 | 2.086 | **3.56 (1.58–8.05)** |
| Smoking (current within 1 mo) | Yes vs No | 2 | 1 | — | -0.012 | 0.779 | -1.538 | 1.514 | **0.99 (0.21–4.54)** |
| Diabetes Mellitus | Yes vs No | 2 | 1 | — | 0.207 | 0.348 | -0.474 | 0.889 | **1.23 (0.62–2.43)** |
| Elective Case | No vs Yes | 1 | 2 | — | 1.980 | 0.720 | 0.569 | 3.390 | **7.24 (1.77–29.67)** |
| Myofascial Release | No vs Yes | 1 | 2 | — | 1.348 | 0.493 | 0.382 | 2.314 | **3.85 (1.47–10.11)** |
| Drains used | No vs Yes | 1 | 2 | — | 0.280 | 0.793 | -1.275 | 1.835 | **1.32 (0.28–6.27)** |
| Concomitant procedure | Yes vs No | 2 | 1 | — | -0.707 | 0.575 | -1.835 | 0.420 | **0.49 (0.16–1.52)** |

**Summary:** Odds of a 1-year recurrence are decreased by 27% for Fo8 vs running suture; not statistically significant (95% CI: 0.36–1.47).

Δ = magnitude of contrast (High − Low) for continuous variables. β = log-odds coefficient. OR = adjusted odds ratio with 95% CI. "Not estimable" indicates convergence failure (typically due to zero/empty cells). Yellow-highlighted row = primary comparison (Fo8 vs Running suture).

**Supplemental Table 10.** Reoperation at 30 Days

| **Variable** | **Contrast** | **Low** | **High** | **Δ** | **β** | **SE** | **95% CI Lower** | **95% CI Upper** | **Odds Ratio (95% CI)** |
| --- | --- | --- | --- | --- | --- | --- | --- | --- | --- |
| Hernia length (cm) |  | 15 | 25 | 10 | 0.181 | 0.189 | -0.190 | 0.552 | **1.20 (0.83–1.74)** |
| Hernia width (cm) |  | 12 | 17 | 5 | 0.189 | 0.104 | -0.014 | 0.392 | **1.21 (0.99–1.48)** |
| BMI |  | 29 | 36.8 | 7.8 | 0.114 | 0.127 | -0.134 | 0.362 | **1.12 (0.88–1.44)** |
| Age |  | 51 | 68 | 17 | 0.233 | 0.163 | -0.087 | 0.554 | **1.26 (0.92–1.74)** |
| **Fascial Closure Technique** | **Fo8 vs Running** | 2 | 1 | — | -0.281 | 0.235 | -0.743 | 0.180 | **0.76 (0.48–1.20)** |
| Procedure: Epigastric (primary ventral) | Yes vs No | 2 | 1 | — | — | 724.800 | — | — | **Not estimable** |
| Procedure: Incisional | No vs Yes | 1 | 2 | — | -0.165 | 1.032 | -2.187 | 1.858 | **0.85 (0.11–6.41)** |
| Recurrent | No vs Yes | 1 | 2 | — | -0.184 | 0.212 | -0.598 | 0.231 | **0.83 (0.55–1.26)** |
| Gender | Male vs Female | 2 | 1 | — | -0.385 | 0.212 | -0.801 | 0.031 | **0.68 (0.45–1.03)** |
| Race: American Indian / Alaskan Native | vs White | 5 | 1 | — | — | 1199.000 | — | — | **Not estimable** |
| Race: Asian, Native Hawaiian / Other Pacific | vs White | 5 | 2 | — | — | 1221.000 | — | — | **Not estimable** |
| Race: Black or African American | vs White | 5 | 3 | — | -0.179 | 0.471 | -1.102 | 0.745 | **0.84 (0.33–2.11)** |
| Race: Hispanic | vs White | 5 | 4 | — | 0.216 | 0.607 | -0.973 | 1.405 | **1.24 (0.38–4.08)** |
| Race: Middle Eastern | vs White | 5 | 6 | — | — | 725.800 | — | — | **Not estimable** |
| Race: Other or unknown | vs White | 5 | 7 | — | 0.407 | 1.033 | -1.618 | 2.431 | **1.50 (0.20–11.38)** |
| Race: Not Indicated | vs White | 5 | 8 | — | — | 869.300 | — | — | **Not estimable** |
| ASA Class: 1 | vs 3 | 3 | 1 | — | — | 615.000 | — | — | **Not estimable** |
| ASA Class: 2 | vs 3 | 3 | 2 | — | -0.358 | 0.291 | -0.928 | 0.211 | **0.70 (0.40–1.24)** |
| ASA Class: 4 | vs 3 | 3 | 4 | — | 0.503 | 0.481 | -0.438 | 1.445 | **1.66 (0.65–4.24)** |
| ASA Class: None Assigned | vs 3 | 3 | 5 | — | — | 1647.000 | — | — | **Not estimable** |
| Immunosuppressants | Yes vs No | 2 | 1 | — | -0.160 | 0.389 | -0.923 | 0.603 | **0.85 (0.40–1.83)** |
| Smoking (current within 1 mo) | Yes vs No | 2 | 1 | — | 0.097 | 0.403 | -0.694 | 0.887 | **1.10 (0.50–2.43)** |
| Diabetes Mellitus | Yes vs No | 2 | 1 | — | 0.106 | 0.228 | -0.341 | 0.554 | **1.11 (0.71–1.74)** |
| Elective Case | No vs Yes | 1 | 2 | — | 0.543 | 0.628 | -0.687 | 1.774 | **1.72 (0.50–5.89)** |
| Myofascial Release | No vs Yes | 1 | 2 | — | 0.142 | 0.369 | -0.581 | 0.865 | **1.15 (0.56–2.38)** |
| Drains used | No vs Yes | 1 | 2 | — | -0.236 | 0.610 | -1.431 | 0.959 | **0.79 (0.24–2.61)** |
| Concomitant procedure | Yes vs No | 2 | 1 | — | -0.101 | 0.288 | -0.666 | 0.465 | **0.90 (0.51–1.59)** |

**Summary:** Odds of a 30-day reoperation are decreased by 25% for Fo8 vs running suture; not statistically significant (95% CI: 0.48–1.20).

Δ = magnitude of contrast (High − Low) for continuous variables. β = log-odds coefficient. OR = adjusted odds ratio with 95% CI. "Not estimable" indicates convergence failure (typically due to zero/empty cells). Yellow-highlighted row = primary comparison (Fo8 vs Running suture).

**Supplemental Table 11.** Reoperation at 1 Year

| **Variable** | **Contrast** | **Low** | **High** | **Δ** | **β** | **SE** | **95% CI Lower** | **95% CI Upper** | **Odds Ratio (95% CI)** |
| --- | --- | --- | --- | --- | --- | --- | --- | --- | --- |
| Hernia length (cm) |  | 16 | 25 | 9 | -0.304 | 0.265 | -0.822 | 0.215 | **0.74 (0.44–1.24)** |
| Hernia width (cm) |  | 12 | 17 | 5 | 0.066 | 0.153 | -0.235 | 0.367 | **1.07 (0.79–1.44)** |
| BMI |  | 28.7 | 36.4 | 7.7 | -0.236 | 0.221 | -0.670 | 0.198 | **0.79 (0.51–1.22)** |
| Age |  | 53 | 68 | 15 | -0.301 | 0.221 | -0.736 | 0.133 | **0.74 (0.48–1.14)** |
| **Fascial Closure Technique** | **Fo8 vs Running** | 2 | 1 | — | -0.070 | 0.362 | -0.780 | 0.639 | **0.93 (0.46–1.90)** |
| Procedure: Epigastric (primary ventral) | Yes vs No | 2 | 1 | — | — | 2628.000 | — | — | **Not estimable** |
| Procedure: Incisional | No vs Yes | 1 | 2 | — | — | 1896.000 | — | — | **Not estimable** |
| Recurrent | No vs Yes | 1 | 2 | — | -0.114 | 0.324 | -0.749 | 0.521 | **0.89 (0.47–1.68)** |
| Gender | Male vs Female | 2 | 1 | — | -0.354 | 0.332 | -1.005 | 0.297 | **0.70 (0.37–1.35)** |
| Race: American Indian / Alaskan Native | vs White | 5 | 1 | — | 2.843 | 1.307 | 0.281 | 5.404 | **17.16 (1.32–222.30)** |
| Race: Asian, Native Hawaiian / Other Pacific | vs White | 5 | 2 | — | — | 5102.000 | — | — | **Not estimable** |
| Race: Black or African American | vs White | 5 | 3 | — | 0.071 | 0.653 | -1.208 | 1.350 | **1.07 (0.30–3.86)** |
| Race: Hispanic | vs White | 5 | 4 | — | — | 1458.000 | — | — | **Not estimable** |
| Race: Middle Eastern | vs White | 5 | 6 | — | — | 2022.000 | — | — | **Not estimable** |
| Race: Other or unknown | vs White | 5 | 7 | — | — | 2476.000 | — | — | **Not estimable** |
| Race: Not Indicated | vs White | 5 | 8 | — | 1.455 | 1.217 | -0.930 | 3.840 | **4.29 (0.40–46.53)** |
| ASA Class: 1 | vs 3 | 3 | 1 | — | — | 2140.000 | — | — | **Not estimable** |
| ASA Class: 2 | vs 3 | 3 | 2 | — | -0.161 | 0.420 | -0.984 | 0.663 | **0.85 (0.37–1.94)** |
| ASA Class: 4 | vs 3 | 3 | 4 | — | 1.224 | 0.607 | 0.034 | 2.414 | **3.40 (1.03–11.18)** |
| Immunosuppressants | Yes vs No | 2 | 1 | — | 0.904 | 0.421 | 0.077 | 1.730 | **2.47 (1.08–5.64)** |
| Smoking (current within 1 mo) | Yes vs No | 2 | 1 | — | 0.150 | 0.642 | -1.109 | 1.408 | **1.16 (0.33–4.09)** |
| Diabetes Mellitus | Yes vs No | 2 | 1 | — | 0.335 | 0.358 | -0.367 | 1.037 | **1.40 (0.69–2.82)** |
| Elective Case | No vs Yes | 1 | 2 | — | 0.724 | 1.114 | -1.459 | 2.907 | **2.06 (0.23–18.30)** |
| Myofascial Release | No vs Yes | 1 | 2 | — | -0.293 | 0.675 | -1.615 | 1.030 | **0.75 (0.20–2.80)** |
| Drains used | No vs Yes | 1 | 2 | — | -0.027 | 1.082 | -2.147 | 2.093 | **0.97 (0.12–8.11)** |
| Concomitant procedure | Yes vs No | 2 | 1 | — | 0.097 | 0.506 | -0.895 | 1.090 | **1.10 (0.41–2.97)** |

**Summary:** Odds of a 1-year reoperation are decreased by 7% for Fo8 vs running suture; not statistically significant (95% CI: 0.46–1.90).

Δ = magnitude of contrast (High − Low) for continuous variables. β = log-odds coefficient. OR = adjusted odds ratio with 95% CI. "Not estimable" indicates convergence failure (typically due to zero/empty cells). Yellow-highlighted row = primary comparison (Fo8 vs Running suture).

**Supplemental Table 12.** HerQLes Score at 30 Days

| **Variable** | **Contrast** | **Low** | **High** | **Δ** | **Estimate** | **SE** | **95% CI Lower** | **95% CI Upper** |
| --- | --- | --- | --- | --- | --- | --- | --- | --- |
| Hernia length (cm) |  | 15 | 25 | 10 | -1.888 | 1.093 | -4.031 | 0.255 |
| Hernia width (cm) |  | 12 | 17 | 5 | -0.846 | 0.649 | -2.118 | 0.427 |
| BMI |  | 29 | 36.8 | 7.8 | -0.700 | 0.762 | -2.195 | 0.795 |
| Age |  | 51 | 68 | 17 | 9.018 | 0.882 | 7.289 | 10.750 |
| **Fascial Closure Technique** | **Fo8 vs Running** | 2 | 1 | — | 1.325 | 1.304 | -1.233 | 3.883 |
| Procedure: Epigastric (primary ventral) | Yes vs No | 2 | 1 | — | -14.770 | 9.861 | -34.100 | 4.571 |
| Procedure: Incisional | No vs Yes | 1 | 2 | — | 3.998 | 4.817 | -5.449 | 13.440 |
| Recurrent | No vs Yes | 1 | 2 | — | 2.661 | 1.165 | 0.375 | 4.946 |
| Gender | Male vs Female | 2 | 1 | — | 3.184 | 1.155 | 0.919 | 5.448 |
| Race: American Indian / Alaskan Native | vs White | 5 | 1 | — | 4.091 | 11.870 | -19.180 | 27.360 |
| Race: Asian, Native Hawaiian / Other Pacific | vs White | 5 | 2 | — | 13.980 | 11.870 | -9.292 | 37.250 |
| Race: Black or African American | vs White | 5 | 3 | — | 2.350 | 2.709 | -2.962 | 7.662 |
| Race: Hispanic | vs White | 5 | 4 | — | 3.493 | 4.403 | -5.142 | 12.130 |
| Race: Middle Eastern | vs White | 5 | 6 | — | 7.715 | 5.951 | -3.955 | 19.390 |
| Race: Other or unknown | vs White | 5 | 7 | — | 15.480 | 6.706 | 2.331 | 28.630 |
| Race: Not Indicated | vs White | 5 | 8 | — | -15.020 | 7.372 | -29.470 | -0.561 |
| ASA Class: 1 | vs 3 | 3 | 1 | — | 17.390 | 6.989 | 3.686 | 31.100 |
| ASA Class: 2 | vs 3 | 3 | 2 | — | 3.531 | 1.481 | 0.626 | 6.435 |
| ASA Class: 4 | vs 3 | 3 | 4 | — | -0.494 | 3.479 | -7.316 | 6.328 |
| ASA Class: None Assigned | vs 3 | 3 | 5 | — | 22.310 | 27.040 | -30.710 | 75.340 |
| Immunosuppressants | Yes vs No | 2 | 1 | — | 5.723 | 1.936 | 1.926 | 9.520 |
| Smoking (current within 1 mo) | Yes vs No | 2 | 1 | — | -6.091 | 2.215 | -10.430 | -1.747 |
| Diabetes Mellitus | Yes vs No | 2 | 1 | — | -0.248 | 1.402 | -2.997 | 2.500 |
| Elective Case | No vs Yes | 1 | 2 | — | 0.214 | 5.614 | -10.800 | 11.220 |
| Myofascial Release | No vs Yes | 1 | 2 | — | 1.654 | 2.616 | -3.475 | 6.784 |
| Drains used | No vs Yes | 1 | 2 | — | 7.421 | 3.510 | 0.538 | 14.300 |
| Concomitant procedure | Yes vs No | 2 | 1 | — | 1.055 | 1.806 | -2.488 | 4.597 |

**Summary:** Fo8 sutures are associated with a 30-day HerQLes score 1.33 points higher than running sutures; not statistically significant (95% CI: −1.23 to 3.88).

Δ = magnitude of contrast (High − Low) for continuous variables. Estimate = adjusted regression coefficient (linear model). Yellow-highlighted row = primary comparison (Fo8 vs Running suture). All effects adjusted for patient and surgical characteristics.

**Supplemental Table 13.** HerQLes Score at 1 Year

| **Variable** | **Contrast** | **Low** | **High** | **Δ** | **Estimate** | **SE** | **95% CI Lower** | **95% CI Upper** |
| --- | --- | --- | --- | --- | --- | --- | --- | --- |
| Hernia length (cm) |  | 17 | 25 | 8 | 0.383 | 1.161 | -1.893 | 2.660 |
| Hernia width (cm) |  | 12 | 17 | 5 | -3.004 | 0.842 | -4.656 | -1.352 |
| BMI |  | 28.9 | 36.3 | 7.4 | 2.218 | 0.965 | 0.325 | 4.112 |
| Age |  | 52 | 68 | 16 | 4.464 | 1.057 | 2.390 | 6.539 |
| **Fascial Closure Technique** | **Fo8 vs Running** | 2 | 1 | — | 0.275 | 1.631 | -2.924 | 3.474 |
| Procedure: Epigastric (primary ventral) | Yes vs No | 2 | 1 | — | 29.200 | 11.550 | 6.540 | 51.860 |
| Procedure: Incisional | No vs Yes | 1 | 2 | — | -7.660 | 6.453 | -20.320 | 4.999 |
| Recurrent | No vs Yes | 1 | 2 | — | 4.303 | 1.490 | 1.380 | 7.226 |
| Gender | Male vs Female | 2 | 1 | — | 4.831 | 1.459 | 1.969 | 7.694 |
| Race: American Indian / Alaskan Native | vs White | 4 | 1 | — | 24.970 | 18.680 | -11.670 | 61.620 |
| Race: Black or African American | vs White | 4 | 2 | — | -2.262 | 3.771 | -9.661 | 5.137 |
| Race: Hispanic | vs White | 4 | 3 | — | -14.250 | 7.149 | -28.280 | -0.229 |
| Race: Middle Eastern | vs White | 4 | 5 | — | 4.374 | 8.062 | -11.440 | 20.190 |
| Race: Other or unknown | vs White | 4 | 6 | — | 4.897 | 10.120 | -14.960 | 24.760 |
| Race: Not Indicated | vs White | 4 | 7 | — | -8.825 | 8.869 | -26.220 | 8.575 |
| ASA Class: 1 | vs 3 | 3 | 1 | — | 6.623 | 6.720 | -6.560 | 19.810 |
| ASA Class: 2 | vs 3 | 3 | 2 | — | 2.242 | 1.863 | -1.413 | 5.897 |
| ASA Class: 4 | vs 3 | 3 | 4 | — | 1.562 | 4.604 | -7.469 | 10.590 |
| Immunosuppressants | Yes vs No | 2 | 1 | — | 1.573 | 2.472 | -3.278 | 6.423 |
| Smoking (current within 1 mo) | Yes vs No | 2 | 1 | — | 1.385 | 3.377 | -5.239 | 8.009 |
| Diabetes Mellitus | Yes vs No | 2 | 1 | — | 0.116 | 1.797 | -3.409 | 3.640 |
| Elective Case | No vs Yes | 1 | 2 | — | -1.517 | 7.021 | -15.290 | 12.260 |
| Myofascial Release | No vs Yes | 1 | 2 | — | -6.651 | 3.281 | -13.090 | -0.214 |
| Drains used | No vs Yes | 1 | 2 | — | -2.098 | 4.630 | -11.180 | 6.984 |
| Concomitant procedure | Yes vs No | 2 | 1 | — | -0.344 | 2.280 | -4.817 | 4.129 |

**Summary:** Fo8 sutures are associated with a 1-year HerQLes score 0.28 points higher than running sutures; not statistically significant (95% CI: −2.92 to 3.47).

Δ = magnitude of contrast (High − Low) for continuous variables. Estimate = adjusted regression coefficient (linear model). Yellow-highlighted row = primary comparison (Fo8 vs Running suture). All effects adjusted for patient and surgical characteristics.

**Supplemental Table 14.** PROMIS Pain 3a T-Score at 30 Days

| **Variable** | **Contrast** | **Low** | **High** | **Δ** | **Estimate** | **SE** | **95% CI Lower** | **95% CI Upper** |
| --- | --- | --- | --- | --- | --- | --- | --- | --- |
| Hernia length (cm) |  | 15 | 25 | 10 | 0.664 | 0.346 | -0.014 | 1.342 |
| Hernia width (cm) |  | 12 | 17 | 5 | 0.138 | 0.206 | -0.267 | 0.542 |
| BMI |  | 29 | 36.8 | 7.8 | 0.095 | 0.241 | -0.379 | 0.568 |
| Age |  | 51 | 68 | 17 | -2.139 | 0.280 | -2.688 | -1.591 |
| **Fascial Closure Technique** | **Fo8 vs Running** | 2 | 1 | — | 0.302 | 0.414 | -0.510 | 1.114 |
| Procedure: Epigastric (primary ventral) | Yes vs No | 2 | 1 | — | 2.391 | 3.159 | -3.803 | 8.586 |
| Procedure: Incisional | No vs Yes | 1 | 2 | — | 0.585 | 1.486 | -2.329 | 3.499 |
| Recurrent | No vs Yes | 1 | 2 | — | -0.174 | 0.370 | -0.900 | 0.551 |
| Gender | Male vs Female | 2 | 1 | — | -2.183 | 0.367 | -2.904 | -1.463 |
| Race: American Indian / Alaskan Native | vs White | 5 | 1 | — | 3.965 | 3.813 | -3.513 | 11.440 |
| Race: Asian, Native Hawaiian / Other Pacific | vs White | 5 | 2 | — | -4.781 | 3.813 | -12.260 | 2.697 |
| Race: Black or African American | vs White | 5 | 3 | — | 1.524 | 0.883 | -0.208 | 3.256 |
| Race: Hispanic | vs White | 5 | 4 | — | -0.997 | 1.398 | -3.738 | 1.744 |
| Race: Middle Eastern | vs White | 5 | 6 | — | -1.554 | 1.912 | -5.304 | 2.196 |
| Race: Other or unknown | vs White | 5 | 7 | — | -4.736 | 1.979 | -8.616 | -0.856 |
| Race: Not Indicated | vs White | 5 | 8 | — | 4.800 | 2.369 | 0.154 | 9.446 |
| ASA Class: 1 | vs 3 | 3 | 1 | — | -4.493 | 2.114 | -8.639 | -0.347 |
| ASA Class: 2 | vs 3 | 3 | 2 | — | -1.249 | 0.473 | -2.176 | -0.321 |
| ASA Class: 4 | vs 3 | 3 | 4 | — | 1.052 | 1.085 | -1.076 | 3.179 |
| ASA Class: None Assigned | vs 3 | 3 | 5 | — | -12.770 | 8.686 | -29.800 | 4.268 |
| Immunosuppressants | Yes vs No | 2 | 1 | — | 0.091 | 0.619 | -1.123 | 1.305 |
| Smoking (current within 1 mo) | Yes vs No | 2 | 1 | — | 2.870 | 0.693 | 1.512 | 4.228 |
| Diabetes Mellitus | Yes vs No | 2 | 1 | — | 0.034 | 0.446 | -0.841 | 0.909 |
| Elective Case | No vs Yes | 1 | 2 | — | -0.511 | 1.765 | -3.973 | 2.950 |
| Myofascial Release | No vs Yes | 1 | 2 | — | 0.879 | 0.829 | -0.746 | 2.504 |
| Drains used | No vs Yes | 1 | 2 | — | -2.001 | 1.133 | -4.222 | 0.221 |
| Concomitant procedure | Yes vs No | 2 | 1 | — | -0.276 | 0.574 | -1.401 | 0.850 |

**Summary:** Fo8 sutures are associated with a 30-day PROMIS pain score 0.30 points higher than running sutures; not statistically significant (95% CI: −0.51 to 1.11).

Δ = magnitude of contrast (High − Low) for continuous variables. Estimate = adjusted regression coefficient (linear model). Yellow-highlighted row = primary comparison (Fo8 vs Running suture). All effects adjusted for patient and surgical characteristics.

**Supplemental Table 15.** PROMIS Pain 3a T-Score at 1 Year

| **Variable** | **Contrast** | **Low** | **High** | **Δ** | **Estimate** | **SE** | **95% CI Lower** | **95% CI Upper** |
| --- | --- | --- | --- | --- | --- | --- | --- | --- |
| Hernia length (cm) |  | 16.8 | 25 | 8.25 | 0.072 | 0.406 | -0.725 | 0.869 |
| Hernia width (cm) |  | 12 | 17 | 5 | 0.497 | 0.285 | -0.063 | 1.056 |
| BMI |  | 28.9 | 36.3 | 7.4 | -0.413 | 0.329 | -1.058 | 0.232 |
| Age |  | 52 | 68 | 16 | -1.818 | 0.360 | -2.524 | -1.111 |
| **Fascial Closure Technique** | **Fo8 vs Running** | 2 | 1 | — | -0.384 | 0.556 | -1.475 | 0.706 |
| Procedure: Epigastric (primary ventral) | Yes vs No | 2 | 1 | — | -6.444 | 3.586 | -13.480 | 0.590 |
| Procedure: Incisional | No vs Yes | 1 | 2 | — | 2.034 | 2.120 | -2.124 | 6.193 |
| Recurrent | No vs Yes | 1 | 2 | — | -0.239 | 0.507 | -1.234 | 0.757 |
| Gender | Male vs Female | 2 | 1 | — | -2.049 | 0.496 | -3.021 | -1.077 |
| Race: American Indian / Alaskan Native | vs White | 5 | 1 | — | 14.370 | 6.376 | 1.865 | 26.880 |
| Race: Asian, Native Hawaiian / Other Pacific | vs White | 5 | 2 | — | -9.540 | 9.034 | -27.260 | 8.183 |
| Race: Black or African American | vs White | 5 | 3 | — | 1.381 | 1.287 | -1.144 | 3.906 |
| Race: Hispanic | vs White | 5 | 4 | — | 7.598 | 2.440 | 2.811 | 12.380 |
| Race: Middle Eastern | vs White | 5 | 6 | — | -3.066 | 2.752 | -8.464 | 2.333 |
| Race: Other or unknown | vs White | 5 | 7 | — | -1.264 | 3.227 | -7.595 | 5.068 |
| Race: Not Indicated | vs White | 5 | 8 | — | -1.363 | 3.027 | -7.302 | 4.576 |
| ASA Class: 1 | vs 3 | 3 | 1 | — | -0.547 | 2.292 | -5.044 | 3.950 |
| ASA Class: 2 | vs 3 | 3 | 2 | — | -1.234 | 0.633 | -2.476 | 0.008 |
| ASA Class: 4 | vs 3 | 3 | 4 | — | -1.085 | 1.570 | -4.165 | 1.996 |
| Immunosuppressants | Yes vs No | 2 | 1 | — | 0.146 | 0.840 | -1.503 | 1.794 |
| Smoking (current within 1 mo) | Yes vs No | 2 | 1 | — | 1.105 | 1.126 | -1.105 | 3.315 |
| Diabetes Mellitus | Yes vs No | 2 | 1 | — | -0.189 | 0.612 | -1.389 | 1.011 |
| Elective Case | No vs Yes | 1 | 2 | — | -2.976 | 2.395 | -7.674 | 1.721 |
| Myofascial Release | No vs Yes | 1 | 2 | — | 2.384 | 1.116 | 0.194 | 4.574 |
| Drains used | No vs Yes | 1 | 2 | — | 1.433 | 1.561 | -1.629 | 4.495 |
| Concomitant procedure | Yes vs No | 2 | 1 | — | -0.076 | 0.779 | -1.605 | 1.454 |

**Summary:** Fo8 sutures are associated with a 1-year PROMIS pain score 0.38 points lower than running sutures; not statistically significant (95% CI: −1.48 to 0.71).

Δ = magnitude of contrast (High − Low) for continuous variables. Estimate = adjusted regression coefficient (linear model). Yellow-highlighted row = primary comparison (Fo8 vs Running suture). All effects adjusted for patient and surgical characteristics.

**Supplemental Table 16.** HRI: Patient-Reported Recurrence (Bulge) at 30 Days

| **Variable** | **Contrast** | **Low** | **High** | **Δ** | **β** | **SE** | **95% CI Lower** | **95% CI Upper** | **Odds Ratio (95% CI)** |
| --- | --- | --- | --- | --- | --- | --- | --- | --- | --- |
| Hernia length (cm) |  | 15 | 25 | 10 | 0.040 | 0.109 | -0.174 | 0.254 | **1.04 (0.84–1.29)** |
| Hernia width (cm) |  | 12 | 17 | 5 | 0.056 | 0.063 | -0.069 | 0.180 | **1.06 (0.93–1.20)** |
| BMI |  | 29 | 36.8 | 7.8 | -0.177 | 0.077 | -0.329 | -0.026 | **0.84 (0.72–0.97)** |
| Age |  | 51 | 68 | 17 | 0.049 | 0.088 | -0.124 | 0.222 | **1.05 (0.88–1.25)** |
| **Fascial Closure Technique** | **Fo8 vs Running** | 2 | 1 | — | -0.012 | 0.130 | -0.266 | 0.242 | **0.99 (0.77–1.27)** |
| Procedure: Epigastric (primary ventral) | Yes vs No | 2 | 1 | — | -0.697 | 1.125 | -2.902 | 1.508 | **0.50 (0.06–4.52)** |
| Procedure: Incisional | No vs Yes | 1 | 2 | — | 0.431 | 0.422 | -0.396 | 1.257 | **1.54 (0.67–3.52)** |
| Recurrent | No vs Yes | 1 | 2 | — | 0.004 | 0.117 | -0.225 | 0.232 | **1.00 (0.80–1.26)** |
| Gender | Male vs Female | 2 | 1 | — | -0.063 | 0.116 | -0.290 | 0.164 | **0.94 (0.75–1.18)** |
| Race: American Indian / Alaskan Native | vs White | 5 | 1 | — | 0.078 | 1.126 | -2.129 | 2.285 | **1.08 (0.12–9.83)** |
| Race: Asian, Native Hawaiian / Other Pacific | vs White | 5 | 2 | — | 0.257 | 1.132 | -1.962 | 2.476 | **1.29 (0.14–11.89)** |
| Race: Black or African American | vs White | 5 | 3 | — | 0.433 | 0.250 | -0.056 | 0.922 | **1.54 (0.94–2.51)** |
| Race: Hispanic | vs White | 5 | 4 | — | 0.275 | 0.415 | -0.538 | 1.088 | **1.32 (0.58–2.97)** |
| Race: Middle Eastern | vs White | 5 | 6 | — | 0.615 | 0.548 | -0.460 | 1.689 | **1.85 (0.63–5.41)** |
| Race: Other or unknown | vs White | 5 | 7 | — | -1.416 | 1.035 | -3.444 | 0.612 | **0.24 (0.03–1.84)** |
| Race: Not Indicated | vs White | 5 | 8 | — | -0.941 | 1.046 | -2.991 | 1.110 | **0.39 (0.05–3.04)** |
| ASA Class: 1 | vs 3 | 3 | 1 | — | -0.093 | 0.671 | -1.408 | 1.222 | **0.91 (0.24–3.39)** |
| ASA Class: 2 | vs 3 | 3 | 2 | — | -0.201 | 0.155 | -0.505 | 0.103 | **0.82 (0.60–1.11)** |
| ASA Class: 4 | vs 3 | 3 | 4 | — | -0.872 | 0.443 | -1.740 | -0.004 | **0.42 (0.18–1.00)** |
| ASA Class: None Assigned | vs 3 | 3 | 5 | — | — | 600.400 | — | — | **Not estimable** |
| Immunosuppressants | Yes vs No | 2 | 1 | — | 0.013 | 0.189 | -0.358 | 0.384 | **1.01 (0.70–1.47)** |
| Smoking (current within 1 mo) | Yes vs No | 2 | 1 | — | 0.127 | 0.210 | -0.284 | 0.538 | **1.14 (0.75–1.71)** |
| Diabetes Mellitus | Yes vs No | 2 | 1 | — | -0.003 | 0.140 | -0.278 | 0.272 | **1.00 (0.76–1.31)** |
| Elective Case | No vs Yes | 1 | 2 | — | 0.385 | 0.531 | -0.656 | 1.425 | **1.47 (0.52–4.16)** |
| Myofascial Release | No vs Yes | 1 | 2 | — | 0.198 | 0.257 | -0.306 | 0.702 | **1.22 (0.74–2.02)** |
| Drains used | No vs Yes | 1 | 2 | — | 0.547 | 0.324 | -0.088 | 1.182 | **1.73 (0.92–3.26)** |
| Concomitant procedure | Yes vs No | 2 | 1 | — | -0.156 | 0.191 | -0.530 | 0.218 | **0.86 (0.59–1.24)** |

**Summary:** Odds of a 30-day patient-reported recurrence are reduced by 1% for Fo8 vs running suture; not statistically significant (95% CI: 0.77–1.27).

Δ = magnitude of contrast (High − Low) for continuous variables. β = log-odds coefficient. OR = adjusted odds ratio with 95% CI. "Not estimable" indicates convergence failure (typically due to zero/empty cells). Yellow-highlighted row = primary comparison (Fo8 vs Running suture).

**Supplemental Table 17.** HRI: Patient-Reported Recurrence (Bulge) at 1 Year

| **Variable** | **Contrast** | **Low** | **High** | **Δ** | **β** | **SE** | **95% CI Lower** | **95% CI Upper** | **Odds Ratio (95% CI)** |
| --- | --- | --- | --- | --- | --- | --- | --- | --- | --- |
| Hernia length (cm) |  | 17 | 25 | 8 | 0.004 | 0.109 | -0.210 | 0.218 | **1.00 (0.81–1.24)** |
| Hernia width (cm) |  | 12 | 17 | 5 | 0.164 | 0.076 | 0.015 | 0.312 | **1.18 (1.02–1.37)** |
| BMI |  | 28.9 | 36.3 | 7.4 | -0.279 | 0.094 | -0.463 | -0.095 | **0.76 (0.63–0.91)** |
| Age |  | 52 | 68 | 16 | -0.131 | 0.100 | -0.327 | 0.064 | **0.88 (0.72–1.07)** |
| **Fascial Closure Technique** | **Fo8 vs Running** | 2 | 1 | — | 0.023 | 0.155 | -0.281 | 0.327 | **1.02 (0.76–1.39)** |
| Procedure: Epigastric (primary ventral) | Yes vs No | 2 | 1 | — | 0.127 | 0.877 | -1.592 | 1.847 | **1.14 (0.20–6.34)** |
| Procedure: Incisional | No vs Yes | 1 | 2 | — | 0.623 | 0.518 | -0.393 | 1.639 | **1.86 (0.68–5.15)** |
| Recurrent | No vs Yes | 1 | 2 | — | -0.191 | 0.143 | -0.472 | 0.090 | **0.83 (0.62–1.09)** |
| Gender | Male vs Female | 2 | 1 | — | -0.065 | 0.139 | -0.337 | 0.207 | **0.94 (0.71–1.23)** |
| Race: American Indian / Alaskan Native | vs White | 5 | 1 | — | — | 671.400 | — | — | **Not estimable** |
| Race: Asian, Native Hawaiian / Other Pacific | vs White | 5 | 2 | — | — | 1442.000 | — | — | **Not estimable** |
| Race: Black or African American | vs White | 5 | 3 | — | 0.867 | 0.306 | 0.267 | 1.466 | **2.38 (1.31–4.33)** |
| Race: Hispanic | vs White | 5 | 4 | — | -0.325 | 0.779 | -1.852 | 1.202 | **0.72 (0.16–3.33)** |
| Race: Middle Eastern | vs White | 5 | 6 | — | 0.755 | 0.643 | -0.506 | 2.015 | **2.13 (0.60–7.50)** |
| Race: Other or unknown | vs White | 5 | 7 | — | -0.820 | 1.095 | -2.965 | 1.326 | **0.44 (0.05–3.77)** |
| Race: Not Indicated | vs White | 5 | 8 | — | 0.169 | 0.817 | -1.432 | 1.770 | **1.18 (0.24–5.87)** |
| ASA Class: 1 | vs 3 | 3 | 1 | — | -0.603 | 0.714 | -2.001 | 0.796 | **0.55 (0.14–2.22)** |
| ASA Class: 2 | vs 3 | 3 | 2 | — | 0.031 | 0.175 | -0.313 | 0.374 | **1.03 (0.73–1.45)** |
| ASA Class: 4 | vs 3 | 3 | 4 | — | 0.098 | 0.417 | -0.719 | 0.915 | **1.10 (0.49–2.50)** |
| Immunosuppressants | Yes vs No | 2 | 1 | — | 0.005 | 0.226 | -0.439 | 0.448 | **1.00 (0.65–1.56)** |
| Smoking (current within 1 mo) | Yes vs No | 2 | 1 | — | 0.237 | 0.297 | -0.345 | 0.819 | **1.27 (0.71–2.27)** |
| Diabetes Mellitus | Yes vs No | 2 | 1 | — | 0.087 | 0.170 | -0.246 | 0.420 | **1.09 (0.78–1.52)** |
| Elective Case | No vs Yes | 1 | 2 | — | -0.036 | 0.681 | -1.370 | 1.298 | **0.96 (0.25–3.66)** |
| Myofascial Release | No vs Yes | 1 | 2 | — | 0.650 | 0.285 | 0.091 | 1.209 | **1.92 (1.09–3.35)** |
| Drains used | No vs Yes | 1 | 2 | — | 0.711 | 0.381 | -0.037 | 1.458 | **2.04 (0.96–4.30)** |
| Concomitant procedure | Yes vs No | 2 | 1 | — | -0.106 | 0.223 | -0.543 | 0.331 | **0.90 (0.58–1.39)** |

**Summary:** Odds of a 1-year patient-reported recurrence are increased by 2% for Fo8 vs running suture; not statistically significant (95% CI: 0.75–1.39).

Δ = magnitude of contrast (High − Low) for continuous variables. β = log-odds coefficient. OR = adjusted odds ratio with 95% CI. "Not estimable" indicates convergence failure (typically due to zero/empty cells). Yellow-highlighted row = primary comparison (Fo8 vs Running suture).
